# ﻿The aquatic Adephaga of the Makay, central-western Madagascar, with description of two new diving beetle species (Coleoptera, Gyrinidae, Haliplidae, Noteridae, Dytiscidae)

**DOI:** 10.3897/zookeys.1127.85737

**Published:** 2022-11-02

**Authors:** Andriamirado Tahina Ramahandrison, Bakolimalala Rakouth, Michaël Manuel

**Affiliations:** 1 Département de Biologie et Ecologie Végétales, Faculté des Sciences, BP906, Université d’Antananarivo, Antananarivo, Madagascar Université d'Antananarivo Antananarivo Madagascar; 2 Sorbonne Université, Institut de Systématique, Evolution, Biodiversité (UMR 7205), MNHN SU CNRS EPHE UA, Case 05, 7 quai St Bernard, Paris, France Sorbonne Université Paris France

**Keywords:** Conservation, *Copelatus*, distribution, diving beetles, endemism, faunistics, forest, freshwater, new species, species diversity

## Abstract

Water beetles of the families Gyrinidae, Haliplidae, Noteridae, and Dytiscidae (aquatic Adephaga) of the Makay in central-western Madagascar were surveyed in three campaigns during the years 2016–2018. A total of 74 species was collected from 62 sampling sites, all except one being newly recorded from the Makay. *Copelatusmalavergnorum***sp. nov.** (*irinus* group) and *C.zanabato***sp. nov.** (*erichsonii* group) (Dytiscidae, Copelatinae) are described and their habitus and male genitalia are illustrated. A systematic account is given, including description of habitat preferences for each species. Analyses of species composition and dominance, species diversity and endemism highlighted the strong singularity of the aquatic Adephaga fauna inhabiting the sandstone massif of inner Makay (notably with several local endemic dytiscids) with respect to its peripheral lowlands. These comparisons were also performed between groups of sites categorised according to vegetation context (forested, semi-forested, non-forested). Rather unexpectedly, inner Makay although well-preserved and little deforested has relatively low endemism level and low species diversity (H_1_ Hill number twice lower than in the geographically close and geologically similar massif of Isalo). Species diversity was higher in the deforested and man-impacted peripheral sites, which yielded a rich contingent of western Madagascar lowland species including a few undescribed or rarely observed dytiscids.

## ﻿Introduction

**Figure 1.**
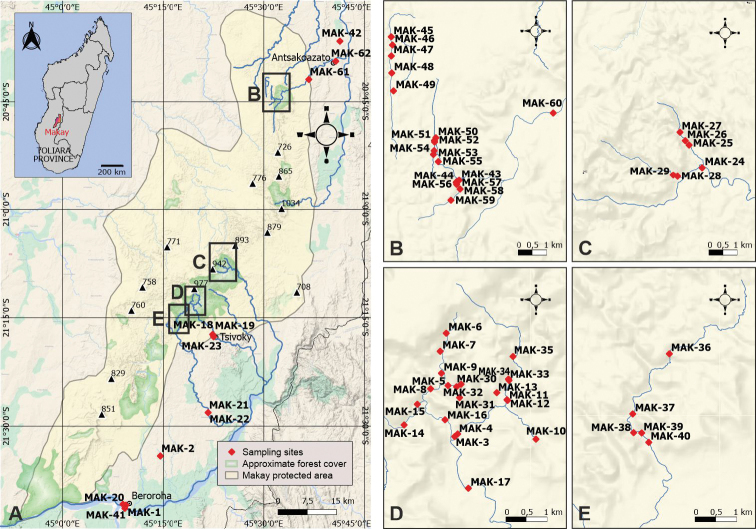
Distribution of sampling sites in the study area **A** map of the Makay protected area, with marked locations of the peripheral sites, and boxes indicating explored areas for inner Makay (top left inset: location of the study area on a map of Madagascar) **B, C, D, E** detailed maps corresponding to the boxes in **A** showing marked locations of the inner Makay sampling sites.

The Makay massif, located in the central-western part of Madagascar (Fig. 1), is one of the most important biodiversity areas of the Island (Carret et al. 2014) and has long remained unexplored. To protect this sanctuary of biodiversity, the status of protected area was granted to the Makay in 2017 (Roubaud et al. 2018). The protected area is bounded to the north by the Malaimbandy municipality and to the south by the Beroroha municipality. With an area of 4000 km^2^, the massif spans 150 km from north to south and 50 km from west to east at its widest. From a cultural point of view, the area encompasses from west to east part of the *Sakalava* country (Menabe region) and of the *Bara* country (Atsimo-Andrefana region). The morphology of the Makay massif is the result of the erosion of the crystalline bedrock (yellow Jurassic sandstone) (Prié 2011), which led to the formation of beautiful and impressive canyons. The regional climate in this part of Madagascar is arid with an average annual rainfall of less than 700 mm (Cornet and Guillaumet 1976), but each canyon presents a number of singularities with respect to microclimatic and pedological conditions, and therefore houses a multitude of micro-habitats. These canyons vary from wide and very sunny to narrow canyons permanently shaded and humid and even to crevices just wide enough to allow water to pass through.

The vegetation is adapted to these contrasting life conditions. At places a typical dense rainforest flourishes, highly similar to that usually found in the eastern part of Madagascar, with the presence of remarkable species such as *Cannarium* trees and arborescent ferns such as *Cyathea*. More open areas are colonised by riparian gallery forest and dense dry forests typical of the western Madagascar ecoregion, dominated by the Fabaceae family (Prié 2011). Microclimate largely depends on the topography of the canyons. Where the slope is very steep and never exposed to direct sunlight, canyon walls can be so damp as to keep a moss carpet and green ferns in all seasons; on the contrary other slopes are so dry that they exhibit a typical saxicolous vegetation dominated by *Pachypodium* and *Uapaca* species. Concerning hydrography, the properties of underground rocks allow them to store water, making the Makay massif the largest freshwater reservoir in western Madagascar. Four large rivers originate from the massif: the Mangoky River to the south, the Maharivo and the Morondava rivers to the west and the Tsiribihina River to the north. As a consequence of these features and of its geographical isolation (the Isalo massif, another Jurassic sandstone massif, is situated at ~ 90 km south of the Makay and separated from it by the large Mangoky River plain), the Makay massif houses a very rich biodiversity and high rates of local endemism, for plants as well as for animals (Wendenbaum 2011; Roubaud et al. 2018).

It was not until 2010 that scientific missions to explore and describe the biodiversity of the Makay started (Wendenbaum 2011; Roubaud et al. 2018). Since then, several multidisciplinary expeditions were organised (Roubaud et al. 2018). Despite some visible signs of habitat degradation resulting from local land use, there is still an exceptional level of ecosystem preservation, and the flora and fauna are highly rich and original. The massif is home to 10 species of lemurs (Dolch et al. 2011; Roubaud et al. 2018). Among the very few other taxa for which more or less systematic species inventories have been conducted in the Makay so far are bats (Prié 2011), scorpions (Lourenço and Wilmé 2015, with description of *Grosphusmakay*), and leafhoppers (Gnezdilov 2021, with description of four new species endemic to the Makay). Additional species recently described from the Makay include an Apocynaceae plant (Allorge et al. 2015), a flea species living on bat (Laudisoit et al. 2012), a fly (Feijen et al. 2021), two millipedes (Wesener 2020) and an ant (Csösz et al. 2021). To our knowledge, there are no published data concerning either Coleoptera or aquatic insects of the Makay apart from the recent description of the endemic diving beetle *Laccophilusmakay* Manuel & Ramahandrison, 2020.

Currently four families and 231 species of aquatic Adephaga (predaceous water beetles) are recorded from Madagascar. The Dytiscidae (in Malagasy, “tsikovoka”) comprise 182 species of which 72% are endemic to Madagascar (however 78% are endemic to the Malagasy region, including Madagascar and the archipelagos of Comoros, Mascarenes, and Seychelles) (Bergsten et al., in press). This family represents the largest portion of the aquatic Adephaga diversity in Madagascar as in the rest of the World. Second in species number is the family Gyrinidae (sister-group to all other Adephaga, Beutel et al. 2020). The Gyrinidae (in Malagasy, “fandiorano”) are represented by 25 recorded species in Madagascar (96% endemic) (Gustafson et al. in press). The family Noteridae comprises 19 species in Madagascar of which 63% are endemic to the country and 68% to the Malagasy region (Bergsten and Manuel, in press). Finally, only six species of Haliplidae are known from Madagascar, all but one endemic to Madagascar and all to the Malagasy region (Bergsten, in press). Members of these last two families are not differentiated by Malagasy people and are often called “tsingala” as for many other aquatic insects (even though this Malagasy word in the strict sense refers to water bugs).

We present here the results of three sampling campaigns targeting aquatic Adephaga, conducted in the Makay area by the authors in June 2016, July-August 2017 and April 2018. A total of 87 samplings was conducted in 62 sampling sites (21 sites in northern Makay and 41 in central-southern Makay, Fig. 1). Of these sites, 50 are located in the massif itself (inner Makay: sandstone and canyons area) and 12 are located in the peripheral plain. The examined material comprises 4151 specimens and 74 species (Gyrinidae: 3; Haliplidae: 1; Noteridae: 8; Dytiscidae: 62), all except *Laccophilusmakay* being newly recorded for the Makay. We consider useful to describe two new species of the genus *Copelatus*, apparently endemic to the massif, because given difficulty of access, new material of these species is not expected to become available soon, and because these species are easily diagnosed thanks to recent revision of the *Copelatus* species of Madagascar (Ranarilalatiana et al. 2019; Ranarilalatiana and Bergsten 2019; Ranarilalatiana et al. in preparation). Distribution and habitat preferences of all species of aquatic Adephaga recorded from the Makay are commented. Special emphasis is put on differences in species composition, species diversity and endemism between the massif and the surrounding plain, and on how the aquatic Adephaga fauna varies in the study area depending on surrounding vegetation (i.e., water bodies located in forested vs. semi-forested or non-forested environment).

## ﻿Materials and methods

### ﻿Abbreviations

**a.s.l.** Above sea level

**E** endemic to Madagascar

E* endemic to the Malagasy region (Madagascar, Seychelles, Comoros, and Mascarene islands)

**F** Forested

**H_0_** Hill number of order q = 0

**H_1_** Hill number of order q = 1

**H_2_** Hill number of order q = 2

**MW** Maximum width

**N** Non forested

pr. Printed

**RFO** Relative frequency of occurrence

**sF** Semi-forested

**TL** Total length

**CMM** Collection of Michaël Manuel, Paris, France

**MNHN**Muséum National d’Histoire Naturelle, Paris, Paris, France

**W** “widespread”, distribution extending beyond the Malagasy region.

### ﻿Depositories

The study specimens are deposited in the last author’s research collection (CMM) and the holotypes of the new species in the MNHN collection.

### ﻿Sampling

Sampling sites are numbered in chronological order of (first) visit from MAK-1 to MAK-62. They are mapped on Fig. 1 and listed below (Sampling data). When several samplings were conducted in the same site (at different sampling dates or in different ecological situations), they are distinguished by adding a letter at the end of the site code (e.g., MAK-1A, MAK-1B: two different samplings performed at site MAK-1). The maps of Fig. 1 were made with QGis 3.22. (https://www.qgis.org) using the 2018 database of the FTM (Foiben-Taosarintanin’i Madagasikara, Institut Géographique et Hygrogaphique National, Antananarivo, Madagascar). The background map was “Google Map Layer”, available in the “XY Layer” menu of QGis.

Three field campaigns were conducted. The first two campaigns (2.–9.VI.2016 and 26.VII.–28.VIII.2017) were conducted in the south-central part of the Makay. In 2016, the area around the Menapanda and the Andranomanintsy rivers was explored (sites MAK-3 to MAK-17, Fig. 1D). In 2017, additional sampling was performed in the same area; furthermore, the canyon of the Makaikely River was visited and more central areas of the massif along the rivers Mahasoa and Behora were targeted (sites MAK-24 to MAK-40, Fig. 1C, D, E). Sampling sites located in the peripheral plain to the south and south-east of the massif were also visited (areas around Beroroha, Tsivoky, and Makaikely), on the way to and from inner Makay, during both campaigns (sites MAK-1, MAK-2, MAK-18 to MAK23, and MAK-41; Fig. 1A). The third field campaign (10.–18.IV.2018) was carried out in the northern part of the Makay along the Sakamaly River and allowed exploration of the Andranomanga and the Ampasimaiky rivers and their surroundings (sites MAK-43 to MAK-60, Fig. 1B). It was noted that the canyons in this northern part of the Makay were drier and wider than in the south-central part. Three sites located in periphery of the massif to the north-east (MAK-42, MAK-61, MAK-62; Fig. 1A), in the areas of Antsakoazato and Tsimazava, were also visited during the 2018 campaign. Collectors were ATR and MM for the 2016 campaign and ATR for the 2017 and 2018 campaigns.

All samplings were performed in situ by hand netting using a GB-net professional hand net (NHBS, Totnes, Devon, UK) (25 cm frame; depth of net bag 50 cm; mesh 1 mm), except at site MAK-22 (light trap).

### ﻿Categories of sampling sites

All sites located in the boxes within the map of Fig. 1A, and whose position is detailed in Fig. 1B–E, were categorised as “inner Makay” (i.e., the Makay massif itself, which we shall refer to also as inner area or canyon area). All sites whose position is detailed in Fig. 1A (thus located outside the boxes) were categorised as “peripheral Makay” (i.e., belonging to the Makay Protected Area but geomorphologically not located in the massif; we shall refer to the corresponding zone as the peripheral area or peripheral plain).

Sites were furthermore categorised according to their vegetation context as determined from field observation completed by inspection of satellite images (Google Earth Pro 7.3.) as “forested”, “semi-forested” or “non-forested”. The context was considered “semi-forested” when a sampling site was located in open or semi-open situation but close to forest edge, or at the bottom of narrow canyons without a proper gallery forest but with a certain density of trees nevertheless present.

### ﻿Sampling data

In the sampling data given below, the letter between parentheses after the sampling code indicates the vegetation context: **F**, forested; **sF**, semi-forested; **N**: non-forested.

**MAK-1A** (N): Beroroha municipality, ca. 2 km W of Beroroa township; ca. 157 m a.s.l.; ca. 21°41'S, 45°09'E; 02.VI.2016; shallow puddles (diameter 1 to 2 meters), with sparse vegetation, along the sandy banks of the Mangoky River.

**MAK-1B** (N): Same as MAK-1A except 09.VI.2016; long and narrow puddle (1 m × 10 m), without vegetation.

**Figure 2.**
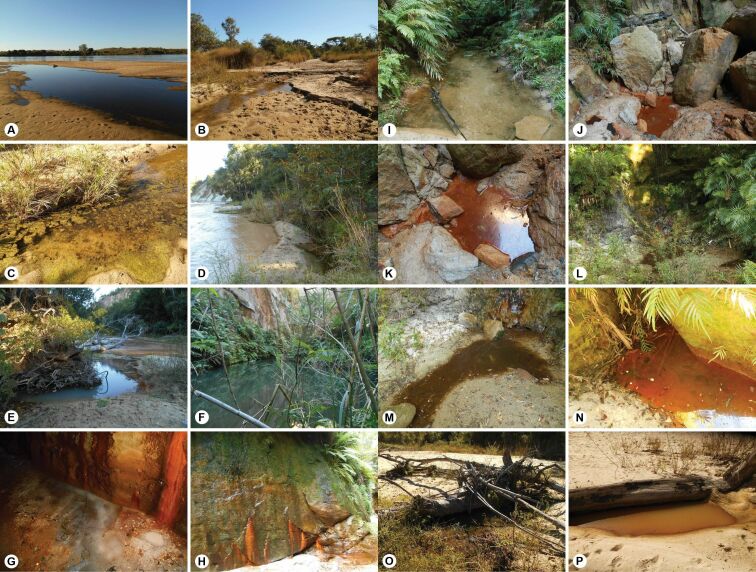
Representative habitats of aquatic Adephaga in Makay. **Sites located in the peripheral area: A** large shallow puddle on the sandy bank of the Mangoky River in Beroroa (MAK-1C), habitat of *Pachynectescostulifer*, *Yolacostipennis***B, C** shallow, slowly flowing stream in semi-open area, with sandy bottom and high density of green algae (MAK-2), habitat of *Bidessuslongistriga*, *B.perexiguus*, *Canthydrusconcolor*, *C.flavosignatus*, *C.guttula*, *Clypeodytesconcivis*, *C.* sp. Ma3, *Cybistercinctus*, *Hydaticusservillianus*, *Hydroglyphusgeminodes*, *Hydrovatusacuminatus*, *H.capnius*, *H.cruentatus*, *H.dentatus*, *H.otiosus*, *H.parvulus*, *H.pictulus*, *H.testudinarius*, *H.* sp. Ma7, *Laccophilusaddendus*, *L.flaveolus*, *L.pallescens*, *L.posticus*, *L.rivulosus*, *L.seyrigi*, *Methles* sp. Ma5, *Neohydrocoptusseriatus*, *Pachynectes* sp. Ma1, *Philaccoluselongatus*, *Pseuduvarus* sp. Ma1, *Rhantaticuscongestus*, *Uvarusrivulorum*. **Sites located in inner Makay: D** puddle (on the right) on the sandy bank of the Andranomanintsy River (left half of the picture) (MAK-3), habitat of *Copelatusruficapillus*, *Hydrovatusacuminatus*, *Hyphydrusseparandus*, *Laccophilusmakay*, *L.posticus*, *Madaglymbusfairmairei*, *Pachynectes* sp. Ma1 **E** spring on the bank of the Andranomanintsy River (MAK-4), habitat of *Copelatusruficapillus*, *Hydroglyphuscapitatus*, *H.geminodes*, *Hyphydrusseparandus*, *Laccophilusmakay*, *L.posticus*, *Pachynectes* sp. Ma1 **F** deep pond above natural dam in the bottom of a canyon (MAK-5A), habitat of *Africophilusnesiotes*, *Hyphydrusseparandus*, *L.addendus*, *Laccophilusinsularum*, *L.makay*, *Neptosternusoblongus*, *Pachynectes* sp. Ma1, *P.* sp. Ma4 **G** edge of small shallow stream, in the bottom of a deep strongly embanked canyon, with orange deposit of iron bacteria (MAK-6), habitat of *Copelatusacamas* and *Laccophilusmakay***H** vertical rock wall with water film and crust of bryophytes and algae, in the bottom of a deep canyon (MAK-9), habitat of *Africophilusbartolozzii***I** large quiet and shaded pool in forest, along streamlet, with masses of tree roots and bottom of sand, gravel and stones (MAK-8), habitat of *Africophilusbartolozzii*, *A.nesiotes*, *Copelatusruficapillus*, *Hydaticussobrinus*, *Hyphydrusseparandus*, *Laccophilusmakay*, *Pachynectes* sp. Ma1 **J** (context) **K** (close-up) Puddle with orange masses of iron bacteria, in stream bed, in the bottom of a deep strongly embanked canyon (MAK-7), habitat of *Copelatusacamas*, *C.ruficapillus*, *Laccophilusmakay***L** (context) **M** (close-up) Small pool with clay-sandy bottom and plant debris, in a small canyon, in gallery forest (MAK-10), habitat of *Copelatusruficapillus*, *Hyphydrusseparandus*, *Laccophilusmakay*, *Madaglymbusfairmairei***N** small shaded pool, with orange masses of iron bacteria, in gallery forest against the wall of a canyon (MAK-14A), habitat of *Africophilusnesiotes*, *Copelatusacamas*, *Hydaticusdorsiger*, *Hyphydrusseparandus*, *Laccophilusmakay*, *Pachynectes* sp. Ma1, *P.* sp. Ma4 **O** small muddy pond, with sparse vegetation, in open area on the sandy banks of the Menapanda River (MAK-11A), habitat of *Canthydrusguttula*, *Copelatuspolystrigus*, *Hydaticusdorsiger*, *Laccophilusaddendus*, *L.posticus*, *Neohydrocoptusseriatus***P** puddle with turbid water and without vegetation, in open area on the sandy banks of the Menapanda River (MAK-11-B), habitat of *Copelatuspolystrigus*, *C.ruficapillus*, *Eretesgriseus*, *Hydaticusdorsiger*, *H.exclamationis*, *Hyphydrusseparandus*, *Laccophilusaddendus*, *L.posticus*, *Madaglymbusfairmairei*.

**MAK-1C** (N): Same as MAK-1B except large shallow puddle (ca. 6 m × 20 m) (Fig. 2A).

**MAK-2** (N): Beroroha municipality, ca. 15 km SW of Makaikely; ca. 245 m a.s.l.; 21°34'08"S, 45°14'32"E; 03.VI.2016; shallow, slowly flowing stream, sandy bottom, with high density of green algae (Fig. 2B, C).

**MAK-3** (sF): Beroroha municipality, ca. 10 km NNW of Tsivoky; ca. 487 m a.s.l.; 21°13'21"S, 45°19'32"E; 04.VI.2016; puddle on the bank of the Andranomanintsy River, sandy bottom; Makay massif (Fig. 2D).

**MAK-4** (sF): Beroroha municipality, ca. 10 km NNW of Tsivoky; ca. 490 m a.s.l.; 21°13'19"S, 45°19'34"E; 04.VI.2016; spring nearby Andranomanintsy River; Makay massif (Fig. 2E).

**MAK-5A** (sF): Beroroha municipality, ca. 10 km NW of Tsivoky; ca. 650 m a.s.l.; 21°12'42"S, 45°19'27"E; 05.VI.2016; deep pond above natural dam in a canyon; Makay massif (Fig. 2F).

**MAK-5B** (sF): same as MAK-5A except slow stream flowing out from the pond, with deep accumulation of organic matter on the bottom.

**MAK-5C** (sF): same as MAK-5A except 17.VIII.2017.

**MAK-5D** (sF): same as MAK-5C except under mass of *Cyathea* roots.

**MAK-6** (N): Beroroha municipality, ca. 10 km NW of Tsivoky; ca. 670 m a.s.l.; 21°12'01"S, 45°19'25"E; 05.VI.2016; quiet corner on the edge of a small stream, in the bottom of a deep strongly embanked canyon, with orange masses of iron bacteria; Makay massif (Fig. 2G).

**MAK-7** (sF): Beroroha municipality, ca. 10 km NW of Tsivoky; ca. 620 m a.s.l.; 21°12'15"S, 45°19'20"E; 05.VI.2016; puddle with orange masses of iron bacteria, in stream bed, in the bottom of a deep strongly embanked canyon; Makay massif (Fig. 2J, K).

**MAK-8** (F): Beroroha municipality, ca. 11 km NNW of Tsivoky; ca. 551 m a.s.l.; 21°12'44"S, 45°19'12"E; 05.VI.2016; large quiet and shaded pool, along streamlet, with masses of tree roots, bottom of sand, gravel and stones; Makay massif (Fig. 2I).

**MAK-9** (N): Beroroha municipality, ca. 11 km NW of Tsivoky; ca. 656 m a.s.l.; 21°12'32"S, 45°19'21"E; 05.VI.2016; vertical rock walls with water film and crust of bryophytes and algae, in the bottom of a deep canyon; Makay massif (Fig. 2H).

**MAK-10** (F): Beroroha municipality, ca. 9 km NNW of Tsivoky; ca. 602 m a.s.l.; 21°13'23"S, 45°20'40"E; 06.VI.2016; small pools with clay-sandy bottom and vegetal debris, in a small canyon; Makay massif (Fig. 2L, M).

**MAK-11A** (N): Beroroha municipality, ca. 10 km NNW of Tsivoky; ca. 514 m a.s.l.; 21°12'53"S, 45°20'16"E; 06.VI.2016; small muddy ponds on sandy bank of the Menapanda River, with sparse vegetation, in open area; Makay massif (Fig. 2O).

**MAK-11B** (N): same as MAK-11A except puddle with turbid water and without vegetation (Fig. 2P).

**MAK-12A** (sF): Beroroha municipality, ca. 10 km NNW of Tsivoky; ca. 516 m a.s.l.; 21°12'53"S, 45°20'16"E; 06.VI.2016; shaded spring on the bank of the Menapanda River, bottom of sand, sandstone mass and decaying vegetal matter, with vegetation and with orange iron bacteria deposit; Makay massif.

**MAK-12B** (sF): same as MAK-12A except 19.VIII.2017.

**MAK-12C** (sF): same as MAK-12A except small pond next to and fed by the spring.

**MAK-13** (F): Beroroha municipality, ca. 10 km NNW of Tsivoky; ca. 527 m a.s.l.; 21°12'47"S, 45°20'07"E; 06.VI.2016; streamlet with vegetation in gallery forest; Makay massif.

**MAK-14A** (F): Beroroha municipality, ca. 10.7 km NW of Tsivoky; ca. 537 m a.s.l.; 21°13'12"S, 45°18'50"E; 07.VI.2016; small shaded pools, with orange masses of iron bacteria, against the walls of a canyon; Makay massif (Fig. 2N).

**MAK-14B** (F): same as MAK-14A, except stream in the bottom of a canyon, bottom of sand and gravel, clear water.

**MAK-15** (F): Beroroha municipality, ca. 10.8 km NW of Tsivoky; ca. 570 m a.s.l.; 21°12'56"S, 45°19'01"E; 07.VI.2016; shallow, shaded stream, clear water, with tree roots; Makay massif.

**MAK-16** (F): Beroroha municipality, ca. 10 km NW of Tsivoky; ca. 506 m a.s.l.; 21°13'08"S, 45°19'24"E; 07.VI.2016; small pond with vegetation, on the bank of the Andranomanintsy River; Makay massif.

**MAK-17** (sF): Beroroha municipality, ca. 8.5 km NW of Tsivoky; ca. 474 m a.s.l.; 21°14'01"S, 45°19'43"E; 08.VI.2016; small isolated puddle, on rock mass, on the bank of the Menapanda River; Makay massif.

**MAK-18** (N): Beroroha municipality, ca. 1 km NW of Tsivoky; ca. 372 m a.s.l.; 21°17'13"S, 45°22'20"E; 08.VI.2016; small and shaded muddy ditch, water rather turbid, no vegetation.

**MAK-19** (N): Beroroha municipality, ca. 800 m NW of Tsivoky; ca. 363 m a.s.l.; 21°17'20"S, 45°22'23"E; 08.VI.2016; large puddle with water slowly flowing, on dirty road between two rice fields, full of rice straw.

**MAK-20** (N): Beroroha municipality, ca. 1,5 km W of Beroroa; ca. 157 m a.s.l.; 21°40'58"S, 45°08'57"E; 09.VI.2016; rice fields near the Mangoky River.

**MAK-21** (N): Beroroha municipality, Makaikely; ca. 243 m a.s.l.; 21°28'8"S, 45°21'41"E; 26.VII.2017; puddle with sandy bottom under *Phragmites*, west bank of the Makaikely River.

**MAK-22** (N): Beroroha municipality, Makaikely; ca. 243 m a.s.l.; 21°28'8"S, 45°21'43"E; 26.VII.2017; light trap.

**MAK-23** (N): Beroroha municipality, Tsivoky; ca. 359 m a.s.l.; 21°17'38"S, 45°22'32"E; 27.VII.2017; Menapanda River near the village of Tsivoky, sandy bottom, with *Cyperus* and *Marsilea*.

**MAK-24** (sF): Beroroha municipality, ca. 18 km NNE of Tsivoky; ca. 484 m a.s.l.; 21°08'2"S, 45°25'4"E; 29.VII.2017; Mahasoa River, sandy bottom; Makay massif.

**MAK-25A** (sF): Beroroha municipality, ca. 19 km NNE of Tsivoky; ca. 501 m a.s.l.; 21°07'36"S, 45°24'48"E; 30.VII.2017; puddle on the sandy banks of the Mahasoa River, with orange deposit of iron bacteria; Makay massif.

**MAK-25B** (sF): same as MAK-25A except small and calm pool under rock along the edge of the river, with tree roots.

**MAK-26** (F): Beroroha municipality, ca. 19 km NNE of Tsivoky; ca. 514 m a.s.l.; 21°07'31"S, 45°24'44"E; 30.VII.2017; quiet part of a stream, bottom of sand and organic matter; Makay massif.

**MAK-27** (F): Beroroha municipality, ca. 19 km NNE of Tsivoky; ca. 526 m a.s.l.; 21°07'22"S, 45°24'37"E; 30.VII.2017; Mahasoa River; Makay massif.

**MAK-28** (sF): Beroroha municipality, ca. 18 km NNE of Tsivoky; ca. 504 m a.s.l.; 21°08'12"S, 45°24'34"E; 01.VIII.2017; small quiet pool in sandy stream bed, water turbid, with accumulation of dead tree leaves; Makay massif.

**MAK-29** (sF): Beroroha municipality, ca. 18 km NNE of Tsivoky; ca. 507 m a.s.l.; 21°08'11"S, 45°24'29"E; 01.VIII.2017; small pool among rocks at the edge of a stream; Makay massif.

**MAK-30** (F): Beroroha municipality, ca. 11 km NNW of Tsivoky; ca. 675 m a.s.l.; 21°12'40"S, 45°19'37"E; 17.VIII.2017; small pool in the bottom of a deep canyon; Makay massif.

**MAK-31A** (sF): Beroroha municipality, ca. 11 km NNW of Tsivoky; ca. 693 m a.s.l.; 21°12'51"S, 45°19'36"E; 17.VIII.2017; small pool among rocks in stream bed; Makay massif.

**MAK-31B** (sF): same as MAK-31A except small deep-water pool in a small cave.

**MAK-31C** (sF): same as MAK-31A except small shaded pool at the entrance of small cave.

**MAK-32** (sF): Beroroha municipality, ca. 11 km NNW of Tsivoky; ca. 650 m a.s.l.; 21°12'42"S, 45°19'34"E; 17.VIII.2017; small pool in the bottom of a canyon, under *Cyathea* tree ferns; Makay massif.

**MAK-33** (F): Beroroha municipality, ca. 10 km NNW of Tsivoky; ca. 525 m a.s.l.; 21°12'37"S, 45°20'17"E; 19.VIII.2017; small puddle with sandy bottom; Makay massif.

**MAK-34A** (F): Beroroha municipality, ca. 10 km NNW of Tsivoky; ca. 538 m a.s.l.; 21°12'36"S, 45°20'16"E; 19.VIII.2017; puddle and spring at the foot of a cliff; Makay massif.

**MAK-34B** (F): same as MAK-34A except: puddle situated more downstream than MAK-34A.

**MAK-35A** (F): Beroroha municipality, ca. 10.5 km NNW of Tsivoky; ca. 536 m a.s.l.; 21°12'20"S, 45°20'21"E; 19.VIII.2017; Small pool among trees, near Menapanda River; Makay massif.

**MAK-35B** (F): same as MAK-35A except puddle in a rock cavity.

**MAK-35C** (F): same as MAK-35A except small stream between MAK-35A and MAK-35B.

**MAK-36A** (F): Beroroha municipality, ca. 10,5 km NW of Tsivoky; ca. 561 m a.s.l.; 21°14'32"S, 45°17'32"E; 21.VIII.2017; streamlet near Andranomanintsy River; Makay massif.

**MAK-36B** (F): same as MAK-36A except small puddle on rock under *Pandanus* tree.

**MAK-37A** (F): Beroroha municipality, ca. 11 km WNW of Tsivoky; ca. 453 m a.s.l.; 21°15'19"S, 45°17'02"E; 24.VIII.2017; water hole in rock mass; Makay massif.

**MAK-37B** (F): same as MAK-37A except very slowly flowing river, bottom of sand and mud, no vegetation.

**MAK-38A** (F): Beroroha municipality, ca. 10.5 km WNW of Tsivoky; ca. 450 m a.s.l.; 21°15'32"S, 45°17'01"E; 25.VIII.2017; small stinky puddle with decaying leaves near Makaikely campment; Makay massif.

**MAK-38B** (F): same as MAK-38A except small pond along river, sandy bottom, water rather turbid, no vegetation.

**MAK-39A** (F): Beroroha municipality, ca. 10.5 km WNW of Tsivoky; ca. 448 m a.s.l.; 21°15'33"S, 45°17'09"E; 25.VIII.2017; small puddle under a *Pandanus* tree, with dead tree leaves; Makay massif.

**MAK-39B** (F): same as MAK-39A except small puddle among rocks along river.

**MAK-40A** (F): Beroroha municipality, ca. 10 km WNW of Tsivoky; ca. 442 m a.s.l.; 21°15'41"S, 45°17'15"E; 25.VIII.2017; small quiet section of a river, sandy bottom, without vegetation; Makay massif.

**MAK-40B** (F): same as MAK-40A except confluence of a small wet zone (located in a depression) with a river.

**MAK-41** (N): Beroroha municipality; ca. 160 m a.s.l.; 21°41'18"S, 45°09'07"E; 28.VIII.2017; small puddle on sandy west bank of River Mangoky.

**MAK-42** (N): Malaimbandy municipality, ca. 5 km NNE of Antsakoazato; ca. 227 m a.s.l.; 20°36'34"S, 45°41'19"E; 10.IV.2018; open marsh, with vegetation of Poaceae, *Cyperus*, *Polygonum* and *Nymphaea*, with water rather turbid and moderate density of filamentous green algae.

**MAK-43** (sF): Malaimbandy municipality, ca. 20 km WSW of Tsimazava; ca. 360 m a.s.l.; 20°44'42"S, 45°31'38"E; 11.IV.2018; shallow margin of the Sakapaly River, water slowly flowing, sandy bottom, with helophytes (Poaceae); Makay massif.

**MAK-44A** (F): Malaimbandy municipality, ca. 20 km WSW of Tsimazava; ca. 364 m a.s.l.; 20°44'42"S, 45°31'35"E; 11.IV.2018; puddle on the east bank of the Sakapaly River, sandy bottom, without organic matter, water red-brown, containing cyanobacteria; Makay massif.

**MAK-44B** (F): same as MAK-44A except bottom with decaying vegetal debris.

**MAK-44C** (F): same as MAK-44A except blind channel connected with the Sakapaly River, with orange masses of iron bacteria on the bottom.

**MAK-45** (sF): Malaimbandy municipality, ca. 21 km W of Tsimazava; ca. 419 m a.s.l.; 20°42'01"S, 45°30'17"E; 12.IV.2018; small pool in a canyon towards Andranomanga; Makay massif.

**MAK-46** (sF): Malaimbandy municipality, ca. 21 km W of Tsimazava; ca. 433 m a.s.l.; 20°42'10"S, 45°30'18"E; 12.IV.2018; pool in a very narrow and dark canyon; Makay massif.

**MAK-47** (sF): Malaimbandy municipality, ca. 21 km W of Tsimazava; ca. 429 m a.s.l.; 20°42'22"S, 45°30'17"E; 12.IV.2018; small pool in a canyon near the Andranomanga River; Makay massif.

**MAK-48** (sF): Malaimbandy municipality, ca. 21 km W of Tsimazava; ca. 451 m a.s.l.; 20°42'41"S, 45°30'18"E; 12.IV.2018; Andranomanga River, water slowly flowing, sandy bottom, no vegetation; Makay massif.

**MAK-49** (sF): Malaimbandy municipality, ca. 21 km W of Tsimazava; ca. 488 m a.s.l.; 20°43'01"S, 45°30'20"E; 12.IV.2018; small pool, bottom of gravel and stones, near the Andranomanga River; Makay massif.

**MAK-50** (sF): Malaimbandy municipality, ca. 20 km WSW of Tsimazava; ca. 437 m a.s.l.; 20°43'54"S, 45°31'10"E; 14.IV.2018; small pond in a canyon at Ampasimaiky; Makay massif.

**MAK-51** (sF): Malaimbandy municipality, ca. 20 km WSW of Tsimazava; ca. 429 m a.s.l.; 20°43'57"S, 45°31'8"E; 14.IV.2018; small pool on dried-out river bed; Makay massif.

**MAK-52** (sF): Malaimbandy municipality, ca. 20 km WSW of Tsimazava; ca. 425 m a.s.l.; 20°43'58"S, 45°31'9"E; 14.IV.2018; sandy pool in canyon along the Ampasimaiky River; Makay massif.

**MAK-53** (sF): Malaimbandy municipality, ca. 20 km WSW of Tsimazava; ca. 423 m a.s.l.; 20°44'8"S, 45°31'8"E; 14.IV.2018; small pool under trees, filled in with tree roots at Ampasimaiky; Makay massif.

**MAK-54A** (sF): Malaimbandy municipality, ca. 20 km WSW of Tsimazava; ca. 418 m a.s.l.; 20°44'12"S, 45°31'7"E; 14.IV.2018; small stream coming out from a canyon at Ampasimaiky; Makay massif.

**MAK-54B** (sF): same as MAK-54A except ca. 416 m a.s.l.; small and slowly flowing derivation of the Ampasimaiky River.

**MAK-55** (sF): Malaimbandy municipality, ca. 20 km WSW of Tsimazava; ca. 409 m a.s.l.; 20°44'20"S, 45°31'13"E; 14.IV.2018; Ampasimaiky River, flowing at the bottom of a canyon; Makay massif.

**MAK-56** (F): Malaimbandy municipality, ca. 20 km WSW of Tsimazava; ca. 366 m a.s.l.; 20°44'44"S, 45°31'34"E; 16.IV.2018; small stream near the Sakapaly River; Makay massif.

**MAK-57** (F): Malaimbandy municipality, ca. 20 km WSW of Tsimazava; ca. 369 m a.s.l.; 20°44'46"S, 45°31'35"E; 16.IV.2018; small water hole filled in with *Pandanus* leaves, near the Sakapaly River; Makay massif.

**MAK-58** (F): Malaimbandy municipality, ca. 20 km WSW of Tsimazava; ca. 377 m a.s.l.; 20°44'51"S, 45°31'39"E; 16.IV.2018; small blind channel on the bank of the Sakapaly River; Makay massif.

**MAK-59A** (F): Malaimbandy municipality, ca. 20 km WSW of Tsimazava; ca. 435 m a.s.l.; 20°45'4"S, 45°31'28"E; 17.IV.2018; quiet part of a stream in Ambilando Canyon, sandy bottom, no vegetation; Makay massif.

**MAK-59B** (F): same as MAK-59A except small pool under a rock mass along the Ambilando stream.

**MAK-59C** (F): same as MAK-59A except Ambilando stream, slow-flowing, sandy bottom with vegetal debris, no vegetation.

**MAK-60** (sF): Malaimbandy municipality, ca. 16 km WSW of Tsimazava; ca. 324 m a.s.l.; 20°43'26"S, 45°33'31"E; 18.IV.2018; open marsh with vegetated margins (Cyperaceae and Polygonaceae), muddy bottom, near the Sakapaly River; Makay massif.

**MAK-61** (N): Malaimbandy municipality, ca. 10 km W of Tsimazava; ca. 286 m a.s.l.; 20°41'53"S, 45°36'41"E; 18.IV.2018; pond along the east bank of the Sakapaly River, muddy bottom, with helophytes (Cyperaceae and Polygonaceae).

**MAK-62** (N): Malaimbandy municipality, Antsakoazato; ca. 235 m a.s.l.; 20°39'21"S, 45°40'42"E; 18.IV.2018; canal at the edge of rice fields, slowly flowing, with muddy bottom, water rather turbid, without vegetation.

### ﻿Morphology and taxonomy

Specimens were morphologically identified to species level by MM (when necessary, with study of dissected genitalia) using the relevant taxonomic literature (reviewed in Bergsten et al. in press) and comparisons with reference specimens in CMM. In difficult cases, type material in the MNHN collection was examined. The nomenclature for Dytiscidae follows the last version of the World Catalogue of Dytiscidae (Nilsson and Hájek 2022). Species which could not be reliably named (i.e., either undescribed species, or species belonging to difficult genera in need of revision, such as *Methles* and *Pseuduvarus*) were assigned a species code (in the form “sp. Ma1”, “sp. Ma2”, etc.).

For illustration of newly described species, photographs of habitus were made with an Olympus SZX12 trinocular stereomicroscope (Tokyo, Japan) using a Spot FLEX Color Pixel Shift 64 Mp camera (Diagnostic Instruments Inc., Sterling Heights, MI, USA) with SPOT BASIC software (http://www.spotimaging.com/software/spot-basic/). For each habitus picture, a Z-series of ~ 30 photos was produced and stacked using HELICON FOCUS Software (Helicon Soft Ltd., Kharkiv, Ukraine), then the surrounding was removed in PHOTOSHOP (Adobe, San Jose, CA, USA) and the image was filtered (Higauss filter, pass 2, strength 1) using the IMAGE PRO PLUS software (Media Cybernetics, Bethesda, MD, USA, http://www.mediacy.com/imageproplus/). Male genitalia were studied and figured in wet condition. Photographs of the genitalia were taken with an Olympus BX61 microscope using a Q imaging camera (15.2 64 Mp Shifting Pixel, Diagnostic Instruments Inc.) with IMAGE PRO PLUS. They were stacked and processed as explained above. The terminology used for genitalia orientation follows Miller and Nilsson (2003). Measurements were made using the “Measure” tool in SPOT BASIC.

Label data of type material are given as written in quotation marks, with separate label lines indicated by a slash (/) and separate labels by a double slash (//). Authors’ additional remarks are provided in square brackets.

### ﻿Analyses of species representativeness and diversity

Relative frequency of occurrence (RFO) of a species for a given set of samplings was calculated by dividing the number of samplings with the species present by the total number of samplings, for the set under consideration.

Interpolation-extrapolation sampling curves (Chao and Jost 2012; Colwell et al. 2012; Chao et al. 2014) were built using iNEXT Online (https://chao.shinyapps.io/iNEXTOnline/) (with default endpoint, 40 nodes and 50 replicates of bootstrap) to quantify and compare species diversity, through estimates of Hill numbers of orders q = 0, q = 1, and q = 2 (respectively noted H_0_, H_1_, and H_2_) across categories of samplings (all; peripheral Makay sites; inner Makay sites; forested sites; semi-forested sites; non-forested sites). For these analyses, numbers of specimens sampled for each species were summed up across samplings associated with each category (see Suppl. material 1: Table S1). For a general explanation about Hill numbers, see Gotelli and Ellison (2013). H_0_ is equivalent to species richness; starting from q=1, the Hill number expresses in “species equivalents” a compromise between species richness and evenness (evenness is maximal if all species present have the same abundances); the higher the order, the higher the weight of evenness with respect to species richness. H_1_ corresponds to the exponential of the classical Shannon index; and H_2_ corresponds to the inverse of the Simpson index.

In order to compare the groups of observations with each other and to quantify similarity/dissimilarity in species composition, Jaccard (based on occurrence data) and Bray-Curtis (based on abundance data) dissimilarity indices were calculated (with the R software). The data were standardised prior to computation of Bray-Curtis indices. The Jaccard dissimilarity index between two sets of objects A and B is equal to 1 - J(A,B) where J(A,B) = |A∩B| / |A∪B|. For the formula of the Bray-Curtis dissimilarity index see Gotelli and Ellison (2013).

## ﻿Results

### ﻿Systematic account

**Family Gyrinidae**

#### ﻿ Dineutus proximus

Taxon classificationAnimaliaColeopteraGyrinidae

Aubé, 1838

C694E792-45DE-5292-855D-FC6797AF2DDC

##### Type locality.

Madagascar.

##### Material examined.

1 ♂, 2 ♀♀: MAK-5B; 1 ♂, 1 ♀: MAK-5D; 2 ♂♂: MAK-13; 2 ♂♂, 3 ♀♀: MAK-14B; 2 ♂♂, 1 ♀: MAK _24; 3 ♂♂, 8 ♀ ♀: MAK-27; 1 ♀: MAK-30; 1 ♀: MAK-35C; 2 ♂♂, 2 ♀♀: MAK-37B; 2 ♀♀: MAK-40A; 2 ♂♂, 3 ♀♀: MAK-40B; 3 ♂♂, 3 ♀♀: MAK-48; 1 ♂, 2 ♀♀: MAK-55; 1 ♂, 1 ♀: MAK-59A; ; 1 ♂, 3 ♀♀: MAK-59C.

##### Distribution.

Madagascar, widespread (Legros 1951; Brinck 1955; Bameul 1984).

##### Habitat in study area.

Collected only in inner Makay, in permanent lotic habitats (rivers and streams) with sandy bottom (in a few sites substrate was more rocky) and with clear water, in forested or semi-forested environmental context, with little or no anthropogenic disturbance.

#### ﻿ Dineutus sinuosipennis sinuosipennis

Taxon classificationAnimaliaColeopteraGyrinidae

Castelnau, 1840

F678DC1E-84F1-534E-A9E0-0CB26991E5BF

= D.bidens Vollenhoven, 1869; D.denticulatus Régimbart, 1882.

##### Type locality.

Tibet (erroneous locality?).

##### Material examined.

1 ♂, 4 ♀♀: MAK-27; 3 ♂♂, 4 ♀♀: MAK-37B; 1 ♂, 1 ♀: MAK-40A; 1 ♂: MAK-40B; 1 ♀: MAK-48; 1 ♂, 5 ♀♀: MAK-52; 5 ♂♂, 9 ♀♀: MAK-55; 1 ♀: MAK-58.

##### Distribution.

Madagascar, widespread (Legros 1951; Brinck 1955; Bameul 1984). Another subspecies, *D.sinuosipenniscomorensis* Régimbart, 1892, is present in the Comoro archipelago (Brinck 1955; Wewalka 1980).

##### Habitat in study area.

Same as *D.proximus* (both species often syntopic). This species is less abundant than *D.proximus* in the Makay massif.

#### ﻿ Orectogyrus vicinus

Taxon classificationAnimaliaColeopteraGyrinidae

Régimbart, 1892

A3183F07-BCE5-5CEB-833F-1F79B65C1A51

##### Type locality.

Madagascar, Diego Suarez (Antsiranana), Isokitra.

##### Material examined.

1 ♂: MAK-15; 2 ♂♂, 3 ♀♀: MAK-36A; 7 ♂♂, 6 ♀♀: MAK-37B; 1 ♀: MAK-40B; 1 ♂, 3 ♀♀: MAK-48.

##### Distribution.

Madagascar. Previously recorded only from the northern part of the island (Legros 1951; Brinck 1956; Gustafson et al. in press).

##### Habitat in study area.

Same as the two preceding species, with a stronger preference for forested and undisturbed habitats.

###### Family Haliplidae

#### ﻿ Peltodytes quadratus

Taxon classificationAnimaliaColeopteraHaliplidae

Régimbart, 1895

A056C291-CD5A-552A-8363-20C951EE8811

##### Type locality.

Madagascar, Antananarivo, Ambodinandohalo Lake.

##### Material examined.

1 ♀: MAK-19; 1 ♂: MAK-41.

##### Distribution.

Madagascar, widespread (Guignot 1959–1961; Bertrand and Legros 1971; Bameul 1984; Rocchi 1991; van Vondel and Bergsten 2012).

##### Habitat in study area.

This species was only found at two sampling sites, both peripheral. One was a large puddle partially sheltered by trees, with water slowly flowing and with abundant rice straw debris, on a dirty road between two rice fields, and the other was a small puddle in open situation on the sandy bank of a river, with *Azolla* aquatic ferns (eutrophication indicator).

**Family Noteridae**

#### ﻿ Canthydrus concolor

Taxon classificationAnimaliaColeopteraHaliplidae

Sharp, 1882

F85261B6-BA41-5FFB-82DA-26242DD476D8

##### Type locality.

Madagascar.

##### Material examined.

1 ♂: MAK-2.

##### Distribution.

Madagascar, widespread (Guignot 1959–1961; Bertrand and Legros 1971; Rocchi 1991; Nilsson 2011).

##### Habitat in study area

**(Fig. 2B, C).** This species is widespread and generally common in Madagascar, in well-vegetated lentic or calm lotic environments. It is seemingly absent from inner Makay, and was sampled only once in a peripheral site. The habitat was a shallow insolated stream characterised by very weak water flow, sandy bottom, marked by anthropic disturbance (cattle trampling), sparse tufts of small Cyperaceae and strong presence of filamentous green algae.

#### ﻿ Canthydrus flavosignatus

Taxon classificationAnimaliaColeopteraHaliplidae

Régimbart, 1903

9C9A5BB1-7ABA-5957-A42C-8E8A74D696B5

##### Type locality.

Madagascar, Fort-Dauphin, Ankara.

##### Material examined.

2 ♂♂: MAK-2; 1 ♀: MAK-19.

##### Distribution.

Zaire (Democratic Republic of the Congo), Madagascar (Guignot 1959–1961; Nilsson 2011).

##### Habitat in study area

**(Fig. 2B, C).** This species was harvested at two sites in the Makay periphery, both in deforested areas. One was the site described for *C.concolor* and the other one was a large puddle, with water slowly flowing and with abundant rice straw debris, on a dirty road between two rice fields.

#### ﻿ Canthydrus guttula

Taxon classificationAnimaliaColeopteraHaliplidae

(Aubé, 1838)

4F3CE9CF-AF9C-5601-851A-980325CF150B

##### Type locality.

La Réunion; Madagascar.

##### Material examined.

3 ♀: MAK-1A; 1 ♀: MAK-2; 1 ♀: MAK-11A; 52 ♂♂, 39 ♀♀: MAK-19; 1 ♂: MAK-20; 1 ♂, 2 ♀♀: MAK-38B; 1 ♂, 3 ♀♀: MAK-40A; 2 ♂♂, 4 ♀♀: MAK-41; 4♂♂, 8 ♀♀: MAK-42; 17 ♂♂, 11 ♀♀: MAK-60; ; 1 ♂: MAK-61.

##### Distribution.

Madagascar and Mascarene Islands; widespread and common in Madagascar (Guignot 1959–1961; Bertrand and Legros 1971; Bameul 1984; Rocchi 1991; Nilsson 2011).

##### Habitat in study area

**(Fig. 2B, C, O).** Collected mainly in peripheral but also in a few inner massif sites. This species is found in permanent or temporary lentic habitats as well as in the calm margins of slowly flowing water bodies, with at least some amount of clay or mud at the bottom. Although a few individuals were taken at some sites without any vegetation or significant accumulation of organic debris, the species is most abundant in well-vegetated habitats and/or with bottom heavily loaded with dead vegetal material. This species has a preference for open environments and is highly tolerant to anthropogenic perturbation (e.g., present in rice fields).

#### ﻿ Canthydrus

Taxon classificationAnimaliaColeopteraHaliplidae

sp. Ma5

4A890A28-1DD4-5A80-A8DF-5EB96F46B5B4

##### Material examined.

1 ♀: MAK-20; 1 ♂, 3 ♀♀: MAK-60; 3 ♀♀: MAK-61.

##### Note.

This species is very similar to *C.flavosignatus* but smaller and with a slightly different shape of the apex of the median lobe of aedeagus in lateral view. The Malagasy species of Noteridae are in great need of revision, and in the current state of knowledge we cannot assign a name to this species.

##### Distribution.

Madagascar. In addition to the specimens from the Makay, we also have specimens from Namoroka (north-eastern part of the island).

##### Habitat in study area.

Species collected in permanent lentic environments in open peripheral sites, with clay bottom, clear water and presence of vegetation. One of the collecting sites was a rice field.

#### ﻿ Neohydrocoptus seriatus

Taxon classificationAnimaliaColeopteraNoteridae

(Sharp, 1882)

85AC78A6-9446-54C3-B348-E88464A8D466

##### Type locality.

Madagascar.

##### Material examined.

2 ♂♂: MAK-2; 1 ♀: MAK-11A; 8 ♂♂, 16 ♀♀: MAK-19; 1 ♂, 2 ♀♀: MAK-21; 20 exs.: MAK-23; 1 ♂: MAK-38B; 2 ♂♂, 1 ♀: MAK-42; 8 ♂♂, 10 ♀♀: MAK-43; 1 ♂, 4 ♀♀: MAK-44A; 1 ♀: MAK-44C; 9 ♂♂, 17 ♀♀: MAK-60; 2 еxs.: MAK-61.

##### Distribution.

Africa (Angola, Guinea, Guinea-Bissau, Mali), Madagascar, and Mascarene Islands (Guignot 1959–1961; Bertrand and Legros 1971; Rocchi 1991; Nilsson 2011). In Madagascar, widespread and common.

##### Habitat in study area

**(Fig. 2B, C, O).** This species is present in lentic and in slowly flowing lotic habitats. It was collected both at peripheral and inner Makay sites. The bottom varied from clay to sandy, with clear, red-brown or turbid water and with more or less abundant plant debris. This species has a clear preference for open environments and habitats with at least some vegetation, and is highly tolerant to anthropogenic disturbance.

#### ﻿ Neohydrocoptus

Taxon classificationAnimaliaColeopteraNoteridae

sp. Ma3

72409EBF-4753-51AA-B71F-9995E6006DB4

##### Material examined.

3 ♂♂, 2 ♀♀: MAK-43; 2 ♀♀: MAK-44C; 4 ♂♂, 1 ♀: MAK-56.

##### Note.

This species is smaller than *N.seriatus* and the elytra do not bear additional serial groups of punctures beyond discal and lateral puncture rows. External features and the shape of the aedeagus evoke *N.aethiopicus* (J. Balfour-Browne, 1961), a widespread sub-Saharan species, but examination of type material in the context of a revision will be necessary to confirm the identity of this species.

##### Distribution.

Madagascar, widespread but not very common.

##### Habitat in study area.

This species was sampled at three sites in inner Makay, in slowly flowing lotic habitats and a dead river arm, in forested or semi-forested contexts without anthropogenic disturbance. These biotopes had sandy bottoms with moderate abundance of plant debris. Two of the sites were surrounded by a well-developed hygrophilous vegetation and contained cyanobacteria.

#### ﻿ Sternocanthus fabiennae

Taxon classificationAnimaliaColeopteraNoteridae

(Bameul, 1994)

85764FA0-4BC6-5B63-A182-738F86AEB2E7

##### Type locality.

Madagascar, Mahajanga, Ambohimanatrika.

##### Material examined.

3 ♂♂, 2 ♀♀: MAK-19; 1 ♂, 1 ♀: MAK-41.

##### Distribution.

Madagascar (Bameul 1994; Nilsson 2011); distribution within the island poorly known.

##### Habitat in study area.

This species was collected at two peripheral sites in lentic habitats in areas with intense anthropogenic pressure: large puddle, with water slowly flowing and with abundant rice straw debris, on a dirty road between two rice fields; and puddle on the sandy banks of the Mangoky River.

#### ﻿ Synchortus asperatus

Taxon classificationAnimaliaColeopteraNoteridae

(Fairmaire, 1869)

23416257-9B8B-5A2F-88A2-C15E174AF292

= S.duplicatus Sharp, 1882.

##### Type locality.

Madagascar.

##### Material examined.

1 ♀: MAK-21; 1 ♂: MAK-42.

##### Distribution.

Madagascar; widespread and common in lowlands (Guignot 1959–1961; Bertrand and Legros 1971; Nilsson 2011).

##### Habitat in study area.

This species was collected at two peripheral sites located in open areas, in temporary lentic habitats without water renewal, and with vegetation.

###### Family Dytiscidae

####### Subfamily Copelatinae, tribe Copelatini

#### ﻿ Copelatus acamas

Taxon classificationAnimaliaColeopteraDytiscidae

Guignot, 1955

2676561C-240C-5DD3-B82B-666BA15DE068

##### Type locality.

Madagascar, Isalo National Parc.

##### Material examined.

1 ♂, 1 ♀: MAK-6; 3 ♂♂: MAK-7; 1 ♀: MAK-14A; 7 ♂♂, 13 ♀♀: MAK-30; 18 ♂♂, 17 ♀♀: MAK-32; 2 ♂♂, 3 ♀♀: MAK-34A; 2 ♂♂, 1 ♀: MAK-34B; 3 ♀♀: MAK-35A; 1 ♂: MAK-39A; 42 ♂♂, 25 ♀♀: MAK-45; 10 ♂♂, 7 ♀♀: MAK-46; 1 ♀: MAK-47; 26 ♂♂, 44 ♀♀: MAK-49; 10 ♂♂, 3 ♀♀: MAK-50; 1 ♀: MAK-52; 11 ♂♂, 5 ♀♀: MAK-53; 1 ♂, 2 ♀♀: MAK-54A; 4 ♂♂, 5 ♀♀: MAK-54B; 48 ♂♂, 57 ♀♀: MAK-59B; 5 ♂♂, 2 ♀♀: MAK-59C.

##### Distribution.

Madagascar; previously known only from the sandstone massif of Isalo (Guignot 1955a).

##### Habitat in study area

**(Fig. 2G, J, K, N).** This species is very common in inner Makay and absent from peripheral sites. It was most often found in puddles and pools located in stream and river beds, as well as small water holes and springs; shaded or sun-exposed, with bottom of sand and/or sandstone, with or without vegetal debris. These water bodies were most often devoid of vegetation. The water was clear but often more or less heavily loaded with orange masses of iron bacteria. Almost all collection points where this species could be observed were in forested or semi-forested areas and all sites were relatively undisturbed.

#### ﻿ Copelatus andobonicus

Taxon classificationAnimaliaColeopteraDytiscidae

Guignot, 1960

4E7E203B-937C-530D-B56C-1C1E935CE1F6

##### Type locality.

Madagascar, Andobo, Antsingy forest.

##### Material examined.

4 ♀: MAK-12A; 2 ♀♀: MAK-33; 1 ♂: MAK-57.

##### Distribution.

Madagascar (Guignot 1960); species characteristic of dry deciduous forests in the western part of the island.

##### Habitat in study area.

This species was collected in lentic habitats (springs and small puddles), with clay or sandy-clay bottom and a lot of plant debris, located in forested or semi-forested areas and relatively unaffected by human disturbances.

#### ﻿ Copelatus polystrigus

Taxon classificationAnimaliaColeopteraDytiscidae

Sharp, 1882

F8647A4E-B6A8-5115-AE8E-6557E98DF456

= C.marginalis Gschwendtner, 1932

##### Type locality.

Madagascar, Senegal.

##### Material examined.

1 ♂: MAK-11A; 1 ♂: MAK-11B; 30 ♂♂, 41 ♀♀: MAK-12A; 1 ♂: MAK-12B; 5 ♂♂, 1 ♀: MAK-12C; 2 ♂♂, 2 ♀♀: MAK-33; 2 ♂♂, 4 ♀♀: MAK-44A; 8 ♂♂, 22 ♀♀: MAK-44B; 23 ♂♂, 22 ♀♀: MAK-44C; 3 ♂♂, 8 ♀♀: MAK-56; 21 ♂♂, 16 ♀♀: MAK-57; 1 ♂: MAK-58.

##### Distribution.

Continental Africa south from Egypt and Sahara, Madagascar (Guignot 1959–1961; Bertrand and Legros 1971). In Madagascar, widespread and common.

##### Habitat in study area

**(Fig. 2O, P).** This species was found in inner Makay, in pools and puddles, mainly in forest. These water bodies were shallow and did not exceed 1 m in depth. The mineral substratum was either clay or sand (or a mixture of both) and there was always a substantial quantity of plant debris.

#### ﻿ Copelatus ruficapillus

Taxon classificationAnimaliaColeopteraDytiscidae

Régimbart, 1895

57D17ECD-3087-5951-860B-4ABBBE4CEAE7

##### Type locality.

Madagascar, Antsiranana, Montagne d’Ambre, Ambohitra National Park.

##### Material examined.

6 ♂♂, 3 ♀♀: MAK-3; 4 ♂♂, 7 ♀♀: MAK-4; 1 ♂: MAK-7; 1 ♀: MAK-8; 6 ♂♂, 10 ♀♀: MAK-10; 1 ♀: MAK-11B; 2 ♂♂, 6 ♀♀: MAK-12A; 2 ♀♀: MAK-12B; 2 ♀♀: MAK-16; 1 ♀: MAK-25A; 1 ♂: MAK-25B; 3 ♂♂, 5 ♀♀: MAK-28; 1 ♂: MAK-29; 1 ♂, 1 ♀: MAK-33; 1 ♀: MAK-34B; 2 ♂♂, 2 ♀♀: MAK-35A; 4 ♂♂: MAK-38A; 1 ♂, 5 ♀♀: MAK-39A; 6 ♂♂, 5 ♀♀: MAK-39B; 1 ♀: MAK-45; 2 ♂♂: MAK-50.

##### Distribution.

Madagascar, widespread (Guignot 1959–1961; Bertrand and Legros 1971).

##### Habitat in study area

**(Fig. 2D, E, I–M, P).** Similar to *C.acamas* (see above).

#### ﻿ Copelatus vigintistriatus

Taxon classificationAnimaliaColeopteraDytiscidae

Fairmaire, 1869

7250C5D6-2596-5E31-98E4-134C4285D58C

##### Type locality.

Mayotte.

##### Material examined.

1 ♀: MAK-44B; 2 ♂♂, 2 ♀♀: MAK-44C; 1 ♀: MAK-56; 2 ♀♀: MAK-60.

##### Distribution.

Madagascar (widespread), Mayotte (Guignot 1959–1961; Bertrand and Legros 1971).

##### Habitat in study area.

This species has been captured in a few inner massif sites all situated in northern Makay: a puddle, a blind channel, a small stream (these sites in forest) and an open marsh. These habitats had slightly turbid water and a mineral bottom ranging from clay to sand with moderate quantity of plant debris, and no vegetation.

#### ﻿ Copelatus malavergnorum

Taxon classificationAnimaliaColeopteraDytiscidae

Manuel & Ramahandrison sp. nov.

0232D3DD-438F-5881-B147-D45537BB1144

https://zoobank.org/C80CC169-845D-438C-A8D4-17939302C057

Figs 3A , 4A–D

##### Type locality.

Madagascar, Toliara province, Malaimbandy municipality, Makay massif (northern part), ca. 20 km WSW Tsimazava, ca. 20°45'S, 45°31'E, altitude ca. 360 m a.s.l.

##### Type material.

***Holotype*** ♂: “Madagascar. Ex-prov. Toliara / Makay massif, ca. 20 km / WSW Tsimazava / 20°44'42"S, 45°31'35"E / 11.IV.2018. Ramahandrison leg. [pr.] // Alt. 364 m. Blind channel / connected with the Sakapaly / River, with orange masses of / iron bacteria on the bottom. [pr.] // Holotype / Copelatusmalavergnorum sp. nov. / Manuel & Ramahandrison, 2022 [red, pr.]” (MNHN).

##### Diagnosis.

This species belongs to the *Copelatusirinus*-group and the *C.insuetus*-complex (revised in Ranarilalatiana et al. 2019). It differs from *C.insuetus* Guignot, 1941 by: smaller size; narrower and more parallel habitus, dorsally flatter; broader pronotum with lateral margins posteriorly more parallel; colour of dorsal and ventral surfaces paler; testaceous basal band of elytron broader; discal stria I on elytron more weakly impressed; strioles on postero-lateral region of pronotum and on metacoxal plate sparser; metacoxal lines shorter; medial lobe of aedeagus in lateral view with apical half of more even width and with apex less narrowly acute, in ventral view distinctly more evenly narrowed from base to apex. This species is externally similar to *C.vokoka* Ranarilalatiana & Bergsten, 2019, but differs by: habitus narrower and more parallel, dorsally flatter; pronotum broader and with lateral margins posteriorly more parallel; discal stria I on elytron more weakly impressed and anteriorly more strongly abbreviated; strioles on pronotum and metacoxal plates much sparser; metacoxal lines shorter; median lobe of aedeagus in lateral view with apical half broader and less strongly arcuate, in ventral view with apex twisted to the left (straight in *C.vokoka*). Finally, it differs from *C.kely* Ranarilalatiana & Bergsten, 2019 notably by the lateral margins of pronotum anteriorly more strongly convergent, the lateral margins of elytra posteriorly more strongly attenuated, the median lobe in lateral view with the distal half much thicker and the dorsal outline less strongly curved, and in ventro-apical view much more gradually narrowed (in *C.kely* abruptly narrowed at ca. midlength from base to apex), subapically thicker and less strongly bent to the left.

##### Description of holotype.

**Figure 3.**
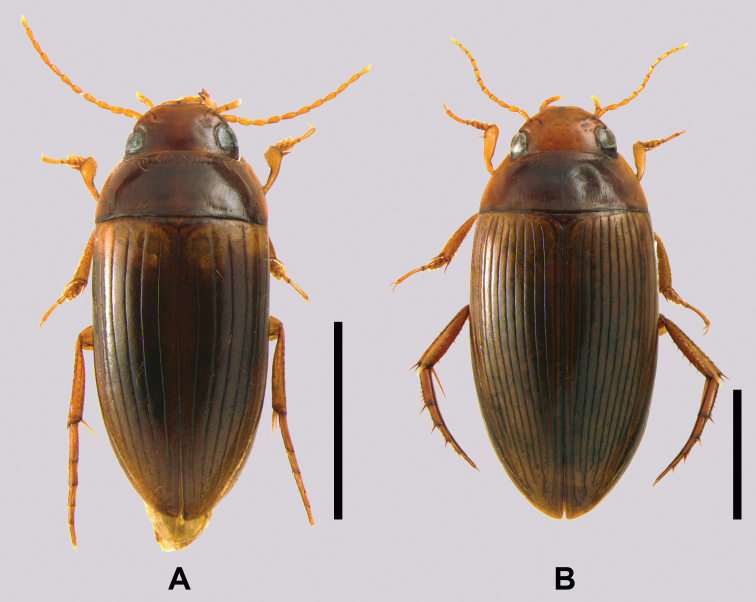
Habitus in dorsal view. Scale bars: 2 mm **A***Copelatusmalavergnorum* sp. nov. (holotype) **B***Copelatuszanabato* sp. nov. (holotype).

Body elongated and parallel-sided (Fig. 3A), weakly convex dorsally. Pronotum broad (ratio between maximum width of pronotum and maximum body width ~ 0.97), with sides posteriorly subparallel. Head rufo-testaceous with only very faint infuscation between eyes. Ratio between interocular distance and maximum width of head ~ 0.66. Pronotum rufo-testaceous as head, with weak medial infuscation. Elytra brown with broad testaceous basal band; testaceous band very diffusely transitioning into darker colour posteriorly (Fig. 3A).

Elytra with six discal and one submarginal striae. Stria I very weakly impressed. Striae I, V, and VI abbreviated anteriorly. Submarginal stria very weakly impressed, starting slightly before elytron midlength. Head, pronotum and elytra with fine reticulation and fine punctation. Posterolateral region of pronotum with few short and weakly impressed strioles.

Ventral side rufo-testaceous, slightly darker laterally on metacoxal plate and on abdominal ventrites. Metacoxal plates with sparse and very fine short strioles; visible abdominal ventrites I-III with denser and longer very fine strioles. Prosternal process rather short and broad, with blunt apex. Metacoxal lines rather long, ending anteriorly at quite small distance from posterior margin of metaventrite, diverging anteriorly.

Appendages: Antennae, palps and legs testaceous. Antennae particularly long (Fig. 3A). First three pro- and mesotarsomeres widened and ventrally equipped with suction cups; number of suction cups per articles (I-III) 7:4:4 on both pro- and mesotarsus. Protibia at base narrow, with bisinuate ventral margin, distally strongly broadened. Pro- and mesotarsal claws unmodified.

**Figure 4.**
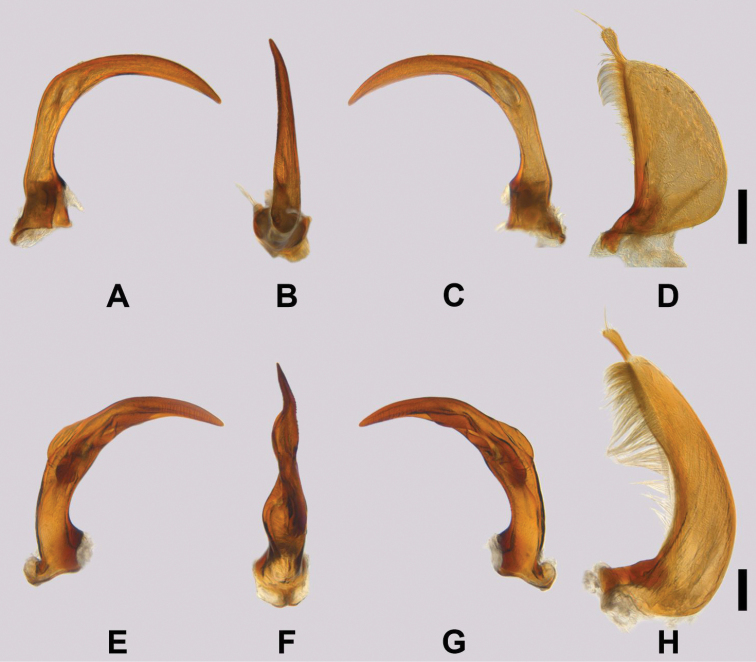
Male genitalia. Scale bars: 200 µm **A–D***Copelatusmalavergnorum* sp. nov. (holotype) **E–H***Copelatuszanabato* sp. nov. (holotype) **A, E** Median lobe of aedeagus in right lateral view **B, F** Median lobe of aedeagus in ventral view **C, G** Median lobe of aedeagus in left lateral view **D, H** Left paramere.

Median lobe and parameres as in Fig. 4A–D.

**Female.** Unknown.

##### Measurements.

TL 4.2 mm, TL without head 3.7 mm, MW 1.8 mm, ratio TL/MW 2.34.

##### Etymology.

This species is dedicated to the Malavergne family (Dominique, Catherine, Clémence, Jacques, and Laurence, Marie-José) in recognition of their constant help and support to the first author during his PhD thesis. The species epithet is a name in the genitive plural.

##### Distribution.

So far known only from northern Makay in Madagascar.

##### Habitat.

The external morphology of this species (very narrow and parallel habitus, broad pronotum, pale colour, long antennae) suggests that it might be a semi-subterranean species. The habitat where the single specimen was found (MAK-44C) was a blind channel connected to River Sakapaly, in northern inner Makay. There was no apparent water flow but the bottom was covered with conspicuous orange masses of iron bacteria, which might be an indication of slow water seepage from underground. The substratum was sandy with moderate amount of decaying vegetal material and the water was red-brown coloured. This water body was fully shaded under trees in forest. There was no vegetation in the water but the surrounding forest floor displayed a typical hygrophilic vegetation of Poaceae, *Cyperus* and *Pandanus*. Other species of aquatic Adephaga (all Dytiscidae) sampled at the same site: *Copelatuspolystrigus*, *C.vigintistriatus*, *Hydrovatusacuminatus* Motschulsky, 1860, *Laccophilusmakay*, *Methles* sp. Ma1, *M.* sp. Ma5, *Neohydrocoptus* sp. Ma3, and *Pachynectes* sp. Ma1.

#### ﻿ Copelatus zanabato

Taxon classificationAnimaliaColeopteraDytiscidae

Manuel & Ramahandrison sp. nov.

DBD337F8-D15C-574D-A2B6-2586C506AB51

https://zoobank.org/7D683E52-D50E-485A-B2CA-5E44D3F4CC5C

Figs 3B , 4E–H

##### Type locality.

Madagascar, Toliara province, Malaimbandy municipality, Makay massif (northern part), ca. 21 km W of Tsimazava, ca. 20°42'S, 45°30'E, altitude ca. 430 m a.s.l.

##### Type material.

***Holotype*** ♂: “Madagascar. Ex-prov. Toliara / Makay massif, ca. 21 km / W Tsimazava / 20°42'10"S, 45°30'18"E [pr.] // 12.IV.2018. Ramahandrison leg. / Alt. 433 m. Pool in a / very narrow and dark / canyon. [pr.] // Holotype / Copelatuszanabato sp. nov. / Manuel & Ramahandrison, 2022 [red, pr.]” (MNHN). Paratypes: 1 ♀: same as holotype. 1 ♂: “Madagascar. Ex-prov. Toliara / Makay massif, ca. 20 km / WSW Tsimazava / 20°43'54"S, 45°31'10"E / 14.IV.2018. Ramahandrison leg. [pr.] // Alt. 437 m. Small / pond in a canyon at / Ampasimaiky [pr.]” (CMM). Both paratypes with respective red label.

##### Diagnosis.

This species belongs to the *Copelatuserichsonii*-group. It is externally rather similar to *C.acamas*, from which it differs by distinctly smaller size; habitus narrower with sides more parallel, dorsally much flatter; discal stria IX on elytron more strongly abbreviated anteriorly; strioles on pronotum surface sparser and much more weakly impressed; shape of median lobe of aedeagus very different. Among *Copelatus* species known from Madagascar, the aedeagus of *C.zanabato* sp. nov. is most similar to that of *C.andobonicus*. From the latter, the new species differs by: habitus narrower with sides more parallel, dorsally much flatter; pronotum paler and elytra with darker linear colouration following the striae much less contrasted with respect to paler background; strioles on pronotum surface denser, present on whole surface (in *C.andobonicus* almost without striae in anterior disk region); median lobe of aedeagus in lateral view with broad flat protuberance on ventral side ca. halfway between base and apex (in *C.andobonicus* with much smaller protuberance at ca. basal third) and apical third much broader, in ventral view with apical region much broader and evenly narrowed, twisted on the left farther from apex.

##### Description of holotype.

Body shape elongate oval, with sides subparallel (Fig. 3B), dorsally weakly convex. Pronotum sides evenly curved and converging from posterior angle. Ratio between maximum width of pronotum and maximum body width ~ 0.90. Head uniformly light rufo-testaceous. Ratio between interocular distance and maximum width of head ~ 0.68. Pronotum medially rufous, laterally colour becoming gradually rufo-testaceous. Elytra light chestnut brown, with darker linear colouration along striae (Fig. 3B).

Elytra with ten well-impressed discal and one submarginal striae. Stria IX abbreviated anteriorly. Striae I and II diverging anteriorly. Submarginal stria starting slightly before elytron midlength, fragmented anteriorly. Head, pronotum and elytra with fine reticulation and fine punctation. Whole surface of pronotum with rather dense, short and fine strioles, in medial disk region strioles even finer.

Ventral side uniformly rufo-testaceous. Metacoxal plates with moderately impressed short strioles; visible abdominal ventrites I-III with denser and longer very fine strioles. Prosternal process short and broad, with rounded apex. Metacoxal lines short, ending anteriorly at large distance from posterior margin of metaventrite, moderately diverging anteriorly.

Appendages: Antennae, palps, forelegs and midlegs testaceous, hindlegs rufo-testaceous. First three pro- and mesotarsomeres widened and ventrally equipped with suction cups; number of suction cups per article (I-III) 7:4:4 on both pro- and mesotarsus. Protibia at base shortly narrow, with shallow protuberance along ventral margin, distally broadened. Pro- and mesotarsal claws unmodified.

Median lobe and parameres as in Fig. 4E–H.

**Female.** Strioles on pronotum surface denser. Pro- and mesotarsomeres and protibia unmodified.

##### Measurements.

Holotype: TL 6.35 mm, TL without head 5.7 mm, MW 2.9 mm, ratio TL/MW 2.20. Paratypes: TL 6.55–6.8 mm, TL without head 5.9–6.1 mm, MW 3.0–3.1 mm, ratio TL/MW 2.17.

##### Variation.

In the male paratype, strioles on pronotum are longer and more deeply impressed than in the holotype, and the metacoxal lines are slightly longer. In the female paratype, the elytral stria V is slightly abbreviated anteriorly.

##### Etymology.

The species name literally means “son of the rock” in Malagasy. It is an invariable name standing in apposition.

##### Distribution.

So far known only from northern Makay in Madagascar.

##### Habitat.

This species was collected at two sites located in two nearby canyons in northern inner Makay. Two specimens were sampled at site MAK-46, an isolated pool (~ 1 m × 3 m) on the bottom of a narrow and dark canyon, and one specimen at site MAK-50, a stagnant temporary pond (~ 3 m × 7 m) situated in a wider canyon and in a more open environment. Both habitats were characterised by sandy bottom with some plant debris, absence of visible inflow / outflow, somewhat turbid water and no vegetation. Other species of aquatic Adephaga (all Dytiscidae) sampled at the same sites: *Copelatusacamas*, *C.ruficapillus*, *Cybisteroperosus* Sharp, 1882, *Hydaticussobrinus* Aubé, 1838, *Hyphydrusseparandus* Régimbart, 1895, *Laccophilusmakay*, *Pachynectes* sp. Ma1, and *P.* sp. Ma4.

#### ﻿ Madaglymbus fairmairei

Taxon classificationAnimaliaColeopteraDytiscidae

(Zimmermann, 1919)

EA4B265D-0BF9-5218-8497-CF3CD498C2BF

= Madaglymbusregimbartii Fairmaire, 1898.

##### Type locality.

Madagascar, Maevatanana.

##### Material examined.

1 ♂, 2 ♀: MAK-3; 2 ♂♂, 8 ♀♀: MAK-10; 1 ♂, 1 ♀: MAK-11B; 2 ♂♂, 2 ♀♀: MAK-12A; 1 ♂: MAK-15; 1 ♂, 1 ♀: MAK-16; 9 ♂♂, 1 ♀: MAK-25A; 3 ♂♂, 1 ♀: MAK-29; 1 ♂: MAK-35A; 1 ♀: MAK-37A; 4 ♂♂, 2 ♀♀: MAK-39A; 1 ♂: MAK-40B.

##### Distribution.

Madagascar (distribution within the island poorly known) (Guignot 1959–1961).

##### Habitat in study area

**(Fig. 2D, L, M, P).** We met this species only in the central part of inner Makay, mainly in puddles and pools with or without water circulation and in small streams, with clear or turbid water, sandy bottom (at some sites with gravel and stones) and moderate to abundant plant debris or tree roots, in forested or semi-forested environments untouched by anthropogenic disturbance.

###### Subfamily Cybistrinae, tribe Cybistrini

#### ﻿ Cybister cinctus

Taxon classificationAnimaliaColeopteraDytiscidae

Sharp, 1882

970BF048-9D89-5243-BB0D-D8AA02D45FC5

##### Type locality.

Madagascar.

##### Material examined.

1 ex.: MAK-1A; 1 ♂, 1 ♂: MAK-2; 1 ♂, 3 ♀♀: MAK-19; 4 ♂♂, 1 еx.: MAK-20; 1 ♀: MAK-23; 1 ♀: MAK-41.

##### Distribution.

Madagascar (widespread and common) (Guignot 1959–1961; Bertrand and Legros 1971; Bameul 1984; Rocchi 1991; Bukontaite et al. 2015).

##### Habitat in study area

**(Fig. 2B, C).** This species was collected only at peripheral sites, in various kinds of water bodies (lentic or slowly flowing) in open areas. It prefers habitats with at least some vegetation and is tolerant to anthropogenic pressure.

#### ﻿ Cybister operosus

Taxon classificationAnimaliaColeopteraDytiscidae

Sharp, 1882

04747A9A-EF84-51F3-9CBE-78770B59079E

##### Type locality.

Madagascar.

##### Material examined.

1 ♀: MAK-50.

##### Distribution.

Madagascar (widespread but localised) (Guignot 1959–1961; Bukontaite et al. 2015).

##### Habitat in study area.

This species was captured only once, in northern inner Makay, in a large temporary pond located in a shallow open area on sand. This single capture does not reflect the usual habitat preferences of this species. According to our observations in Isalo and Ankarafantsika, this is a lotic species (an exceptional ecology for the genus) inhabiting the margins of streams and rivers with some vegetation and / or tree roots and / or plant debris, in well-preserved forested or semi-forested environments.

###### Subfamily Dytiscinae, tribe Aciliini

#### ﻿ Rhantaticus congestus

Taxon classificationAnimaliaColeopteraDytiscidae

(Klug, 1833)

FFF287BE-10D7-5137-BFAB-E5FC147FA128

= R.rochasi (Perroud & Montrouzier, 1864); R.signatipennis (Laporte, 1835).

##### Type locality.

Madagascar.

##### Material examined.

1 ♂: MAK-2; 2 ♀♀: MAK-19; 1 ♂: MAK-41.

##### Distribution.

From sub-Saharan Africa to Australia through tropical Asia and the Oriental region (Guignot 1959–1961; Bertrand and Legros 1971; Watts 1978; Bameul 1984; Hendrich et al. 2010). In Madagascar, widespread.

##### Habitat in study area

**(Fig. 2B, C).** This species was found at three sampling sites in the periphery of the Makay massif: a large puddle partially sheltered by trees, with water slowly flowing and with abundant rice straw debris, on a dirty road between two rice fields; a small puddle in open situation on the sandy bank of a river, with *Azolla* aquatic ferns (eutrophication indicator); and a shallow isolated stream characterised by very weak water flow, sandy bottom, sparse tufts of small Cyperaceae and strong presence of filamentous green algae. These habitats were all situated in open environments and more or less affected by anthropogenic disturbance.

###### Tribe Eretini

#### ﻿ Eretes griseus

Taxon classificationAnimaliaColeopteraDytiscidae

(Fabricius, 1781)

4EF79FFA-0229-5A3F-8987-AEBEF0A7CE01

= E.plicipennis (Motschulsky, 1845); E.succinctus (Klug, 1834).

##### Type locality.

India.

##### Material examined.

1 ♂, 1 ♀: MAK-11B.

##### Distribution.

Southern half of the Palearctic region, Africa, Oriental region (Guignot 1959–1961; Miller 2002). In Madagascar, widespread.

##### Habitat in study area

**(Fig. 2P).** This species was collected at a single site in central inner Makay, in an isolated shallow temporary puddle located in the middle of a large flat accumulation of sand in the outer part of river meander. The environment was totally open with no trees, the water was turbid and there was no vegetation. These habitat characteristics are very representative of the ecology of members of the genus *Eretes* everywhere in the world (Miller 2002).

###### Tribe Hydaticini

#### ﻿ Hydaticus dorsiger

Taxon classificationAnimaliaColeopteraDytiscidae

Aubé, 1838

9D6C4C3E-08FE-5BAC-9EA6-FAE4AED12CDF

##### Type locality.

Madagascar.

##### Material examined.

1 ♂: MAK-11A; 1 ♀: MAK-11B; 1 ♂, 1 ♀: MAK-12A; 1 ♂: MAK-14A; 1 ♂: MAK-18; 8 ♂♂, 6 ♀♀: MAK-19; 1 ♂: MAK-23.

##### Distribution.

Whole tropical Africa to Arabia, Madagascar (where it is widespread and common) (Guignot 1959–1961; Bertrand and Legros 1971; Bameul 1984; Rocchi 1991; Hájek and Reiter 2014; Bukontaite et al. 2015).

##### Habitat in study area

**(Fig. 2N–P).** This species has been observed mainly at peripheral sites and in open areas, in lentic environments that were permanent or temporary, with or without slow water flow, with bottom often muddy, and with or without marginal vegetation. This species is tolerant to anthropogenic disturbance.

#### ﻿ Hydaticus exclamationis

Taxon classificationAnimaliaColeopteraDytiscidae

Aubé, 1838

D4413859-D124-5668-B492-926123E7AB90

##### Type locality.

Madagascar.

##### Material examined.

1 ♂: MAK-11B.

##### Distribution.

Sub-Saharan Africa, Mauritius, Madagascar (Guignot 1959–1961; Bameul 1984; Bukontaite et al. 2015). In Madagascar, widespread and common, especially in lowlands.

##### Habitat in study area

**(Fig. 2P).** This species was collected at a single site in south-central inner Makay (see description of the habitat above under *Eretesgriseus*). It usually prefers lentic or slowly flowing habitats with at least some vegetation.

#### ﻿ Hydaticus petitii

Taxon classificationAnimaliaColeopteraDytiscidae

Aubé, 1838

FAD911BC-BCBA-561F-9F1E-800B170F82CE

##### Type locality.

Madagascar.

##### Material examined.

1 ♂: MAK-52.

##### Distribution.

Madagascar, widespread (Guignot 1959–1961; Bukontaite et al. 2015).

##### Habitat in study area.

This species was collected once, in northern inner Makay, in a calm pool (~ 3 m × 7 m) on the bottom of a canyon, with inflow and outflow from the nearby Ampasimaiky River. This pool was rather deep (> 1 m), with bottom of sand covered with a thin layer of clay, almost without vegetal detritus, with moderately turbid water and no vegetation. The environment was semi-forested and the pond was surrounded by *Ravenea* palm trees. As a rule this Malagasy endemic species is encountered in forest massifs in more or less undisturbed habitats.

#### ﻿ Hydaticus servillianus

Taxon classificationAnimaliaColeopteraDytiscidae

Aubé, 1838

C64E2894-9457-5290-A427-1FBA2FDB2B2D

= H.discoidalis Hope, 1843; H.flavomarginatus Zimmermann, 1920).

##### Type locality.

South Africa, Western Cape, Cape of Good Hope.

##### Material examined.

1 ♀: MAK-1A; 1 ♂: MAK-2; 13 ♂♂, 7 ♀♀: MAK-19; 1 ♂, 3 ♀♀: MAK-23; 2 ♂♂: MAK-40A; 1 ♂, 6 ♀♀: MAK-61; 1 ♂: MAK-62.

##### Distribution.

Sub-Saharan Africa, Madagascar (Guignot 1959–1961; Bameul 1984; Hájek and Reiter 2014; Bukontaite et al. 2015).

##### Habitat in study area

**(Fig. 2B, C).** This species has been found in peripheral sites and at one inner massif site, in lentic and slowly flowing lotic habitats, mainly in open areas. The bottom comprised various amounts of sand and clay/mud and moderate to abundant plant debris. The water was clear to moderately turbid. The vegetation was variously developed. This eurytopic species is tolerant to anthropogenic perturbation.

#### ﻿ Hydaticus sobrinus

Taxon classificationAnimaliaColeopteraDytiscidae

Aubé, 1838

10EFC99D-F030-5636-9E57-0F45361FB166

= Hydaticusmatruelisvar.obliquevittatus Régimbart, 1895).

##### Type locality.

Madagascar, Mascarene Islands (Guignot 1959–1961; Bertrand and Legros 1971; Bameul 1984; Bukontaite et al. 2015).

##### Material examined.

1 ♀: MAK- 8; 2 ♀: MAK-50; 2 ♀♀: MAK-53; 1 ♂: MAK-54A; 2 ♀♀: MAK-59C.

##### Distribution.

Mauritius, La Réunion, Madagascar. In Madagascar, widespread.

##### Habitat in study area.

This species was collected only in inner Makay, in well-preserved forested or semi-forested areas. The habitats were isolated pools and very slowly flowing streams, with clear or slightly turbid water, sandy bottom (sometimes with stones), with moderate amount of plant debris and no vegetation.

###### Subfamily Hydroporinae, tribe Bidessini

#### ﻿ Bidessus longistriga

Taxon classificationAnimaliaColeopteraDytiscidae

Régimbart, 1895

B0BE2A6E-37BF-5061-A48F-48FF7ACE6681

##### Type locality.

Madagascar, Antsiranana,

##### Material examined.

1 ♀: MAK-1A; 1 ♂: MAK-2; 2 ♀♀: MAK-19; 17 еxs.: MAK-42; 1 еx.: MAK-60; 1 еx.: MAK-61.

##### Distribution.

Madagascar, widespread (Guignot 1959–1961; Bertrand and Legros 1971; Bameul 1984; Biström 1985; Rocchi 1991; Bergsten et al. 2020).

##### Habitat in study area

**(Fig. 2B, C).***Bidessuslongistriga* was sampled at peripheral sites and at one inner massif site, in open areas, in permanent or temporary lentic environments, with water stagnant or slowly flowing, clear or turbid, with sand and/or clay bottom. It prefers habitats with at least some marginal vegetation, and is tolerant to anthropogenic disturbance.

#### ﻿ Bidessus perexiguus

Taxon classificationAnimaliaColeopteraDytiscidae

H. J. Kolbe, 1883

8BC18AA8-D984-5DC7-89C9-CEA4DA688318

##### Type locality.

Madagascar, South inner part.

##### Material examined.

1 ex.: MAK-1A; 4 ♂♂, 1 ex.: MAK-2; 1 ♂: MAK-17; 82 exs.: MAK-18; 4 ♂♂, 25 exs.: MAK-19; 3 exs.: MAK-60; 1 ex.: MAK-61.

##### Distribution.

Madagascar, widespread (Guignot 1959–1961; Biström 1985; Bergsten et al. 2020).

##### Habitat in study area

**(Fig. 2B, C).** This species was collected mainly at peripheral sites. Its ecology is similar to that of *B.longistriga*, and both species were several times sampled in the same habitats, but *B.perexiguus* has a marked preference for small and very shallow water bodies without vegetation. Notably, this species was particularly abundant in a small muddy ditch only 5 cm deep, shaded under trees, without water flow and with turbid water, trampled by cattle and with no vegetation (MAK-18).

#### ﻿ Clypeodytes concivis

Taxon classificationAnimaliaColeopteraDytiscidae

Guignot, 1955

2B4F3095-6035-530D-8825-99A222775A7E

##### Type locality.

Madagascar, Iharanandriana mountain.

##### Material examined.

3 ♂♂, 3 ♀♀: MAK-2; 1 ♂: MAK-61.

##### Distribution.

Madagascar, widespread (Guignot 1955b; Biström 1988a).

##### Habitat in study area

**(Fig. 2B, C).** This species was collected at two peripheral sites, a very slowly flowing and shallow stream, and a shallow stagnant pond, both in open environments. The mineral substratum was a mixture of sand and clay in the first case, and of clay and mud in the second case, with moderate amount of plant debris. The water was clear and there was a marginal vegetation of helophytes; at site MAK-2 with abundant filamentous green algae. At both sites, the biotope was trampled and enriched in nutrients by cattle.

#### ﻿ Clypeodytes insularis

Taxon classificationAnimaliaColeopteraDytiscidae

Guignot, 1956

8439E0F8-FDC4-535B-8F9E-86129F95C634

##### Type locality.

Madagascar, Bas Mangoky agricultural station.

##### Material examined.

1 ♂: MAK-20.

##### Distribution.

Madagascar, widespread but not common (Guignot 1956; Biström 1988a)

##### Habitat in study area.

A single specimen of this species was taken in a rice field on the banks of the Mangoky River (a peripheral site), at shallow depth. The bottom was composed of sand and clay with some plant debris; there was no visible inlet or outlet, the water was clear and there was a moderate presence of green algae. The environment was open with no trees in the surroundings.

#### ﻿ Clypeodytes

Taxon classificationAnimaliaColeopteraDytiscidae

sp. Ma3

3442DAA6-A3BA-514C-A2D6-2B37E799A179

##### Material examined.

1 ♂: MAK-2.

##### Distribution.

Madagascar (known to us only from site MAK-2).

##### Note.

This is an undescribed species, close to *C.spangleri* Biström, 1988 and *C.pseudolentus* Biström, 1988 from continental Africa.

##### Habitat in study area

**(Fig. 2B, C).** This species was sampled only once in a peripheral site. The habitat was a shallow isolated stream characterised by very weak water flow, sandy bottom, marked anthropic disturbance (cattle trampling), sparse tufts of small Cyperaceae and strong presence of filamentous green algae.

#### ﻿ Hydroglyphus capitatus

Taxon classificationAnimaliaColeopteraDytiscidae

(Régimbart, 1895)

2978C872-5947-596A-8097-5C7677064E6E

= H.longivittis Régimbart, 1903.

##### Type locality.

Madagascar, Antsiranana.

##### Material examined.

13 ♂♂, 20 ♀: MAK-1A; 1 ♂, 1 ♀: MAK-4; 5 ♂♂: MAK-61.

##### Distribution.

Seychelles, Madagascar (Guignot 1959–1961; Biström 1986; Rocchi 1991). In Madagascar, widespread and common particularly in lowlands.

##### Habitat in study area

**(Fig. 2E).** This species was captured at two peripheral and at one inner massif sites, in highly contrasted habitats, including shallow puddles on the sandy banks of the Mangoky River, a shallow pond with clay-mud bottom and marginal helophytes, and (for the inner Makay site) a marginal spring on the bank of a river, full of orange masses of iron bacteria. The environment was open and strongly impacted by human activities in the two peripheral sites (where the species was more abundant); semi-forested and rather well preserved in the inner massif site.

#### ﻿ Hydroglyphus geminodes

Taxon classificationAnimaliaColeopteraDytiscidae

(Régimbart, 1895)

DB0D9FC7-C9CF-5BF1-9614-04D6F65A8487

= H.africanus Régimbart, 1895.

##### Type locality.

Madagascar, Antsiranana.

##### Material examined.

6 ♂♂, 3 ♀: MAK-1A; 1 ♂, 2 ♀: MAK-2; 6 ♂♂, 3 ♀♀: MAK-4; 2 ♂♂, 10 ♀♀: MAK-17; 1 ♂: MAK-18; 4 ♂♂, 3 ♀♀: MAK-19; 4 ♂♂, 1 ♀: MAK-61.

##### Distribution.

Sub-Saharan Africa, Mauritius, La Réunion, Madagascar (Guignot 1959–1961; Bertrand and Legros 1971; Bameul 1984; Biström 1986; Rocchi 1991). In Madagascar, widespread and common.

##### Habitat in study area

**(Fig. 2B, C, E).** This species has been found mainly at peripheral sites in open areas, at shallow depth in various kinds of water bodies (temporary or permanent lentic habitats with water stagnant or very slowly flowing). The water was generally clear and the bottom consisted of sand and/or clay, with some plant debris. There was either no vegetation or sparse helophytes and at some sites presence of filamentous green algae. This species is tolerant to anthropogenic disturbance.

#### ﻿ Hydroglyphus plagiatus

Taxon classificationAnimaliaColeopteraDytiscidae

(H. J. Kolbe, 1883)

46B80720-81F8-5E9C-8EA8-1ED7D63CEC68

##### Type locality.

Eastern part of Madagascar.

##### Material examined.

1 ♂: MAK-17.

##### Distribution.

Madagascar, common in the Central Highlands (Guignot 1959–1961; Bertrand and Legros 1971; Bameul 1984, Biström 1986; Rocchi 1991).

##### Habitat in study area.

This species seems very rare in the Makay since only one specimen was found, in an inner massif site located in a semi-forested environment. The habitat was a small, isolated, sun-exposed puddle on rock mass, on the bank of a river. The bottom consisted of sandstone, sand and clay without organic debris, the water was clear and there was no vegetation.

#### ﻿ Liodessus luteopictus

Taxon classificationAnimaliaColeopteraDytiscidae

(Régimbart, 1897)

370740EA-6912-5849-8481-CB483889DB19

= L.poecilopterus Régimbart, 1900.

##### Type locality.

Mascarene Islands, Mauritius, Curepipe.

##### Material examined.

1 ♂: MAK-16.

##### Distribution.

Mauritius, La Réunion, Comoros, Madagascar (Guignot 1959–1961; Bameul 1984; Biström 1988b). In Madagascar, widespread.

##### Habitat in study area.

This species was sampled at a single inner massif site, a small pond, partly shaded, at the edge of a gallery forest close to River Andranomanintsy. The water was slowly renewed from the nearby river, the bottom was sandy with moderate plant debris and the water was clear. This small water body was filled in with subaquatic Poaceae including a *Panicum* species.

#### ﻿ Pachynectes costulifer

Taxon classificationAnimaliaColeopteraDytiscidae

(Régimbart, 1903)

154AA14C-A161-5C72-90B9-BB0864919B02

##### Type locality.

Madagascar, Imanombo.

##### Material examined.

4 ♂♂, 2 ♀♀: MAK-1A; 3 ♂♂, 11 ♀♀: MAK-1B; 12 ♂♂, 26 ♀♀: MAK-1C; 2 ♂♂: MAK-20; 2 ♂♂: MAK-22.

##### Distribution.

Madagascar, western and southern parts of the island (Guignot 1959–1961; Bertrand and Legros 1971; Biström 1987; Rocchi 1991).

##### Habitat in study area

**(Fig. 2A).** This species was found only at peripheral sites located on the large sandy banks of the Mangoky River close to Beroroha, in small to large shallow isolated puddles, with sand or sand-clay bottom and clear water, without vegetation or with sparse vegetation, with or without presence of filamentous green algae.

#### ﻿ Pachynectes

Taxon classificationAnimaliaColeopteraDytiscidae

sp. Ma1

59D02A6C-4DF9-57F3-98F2-D27E3455827A

##### Material examined.

5 ♂♂, 2 ♀♀: MAK-2; 25 ♂♂, 22 ♀♀: MAK-3; 8 ♂♂, 4 ♀♀: MAK-4; 3 ♂♂, 1 ♀: MAK-5A; ; 8 exs.: MAK-5D; 2 ♀♀: MAK-8; 29 ♂♂, 14 ♀♀: MAK-14A; 5 ♂♂, 5 ♀♀: MAK-16; 24 exs.: MAK-28; 24 exs.: MAK-29; 3 exs.: MAK-36B; 91 exs.: MAK-37A; 3 exs: MAK-38B; 53 exs.: MAK-40A; 1 ex.: MAK-43; 1 ex.: MAK-44C; 2 exs.: MAK-45; 28 exs.: MAK-47; 5 exs.: MAK-49; 31 exs.: MAK-50; 15 exs.: MAK-51; 37 exs.: MAK-52; 10 exs.: MAK-53; 2 exs.: MAK-54B; 6 exs.: MAK-58; 39 exs.: MAK-59A; 25 exs.: MAK-59C.

##### Note.

This is a probably undescribed species close to *P.hygrotoides* (Régimbart, 1895). The Malagasy endemic genus *Pachynectes* is currently being revised (J. Bergsten, pers. comm.) and many species are awaiting description.

##### Distribution.

Madagascar. The exact distribution of this species within the island remains to be established in the context of the upcoming revision, but it is not endemic to the Makay (sampled by us notably in the Isalo Massif).

##### Habitat in study area

**(Fig. 2B–F, I, N).** This is one of the most abundant species of aquatic Adephaga in inner Makay, and it was also collected at one peripheral site (MAK-2). It was sampled in all kinds of lentic or very slowly flowing lotic habitats (puddles, pools, ponds, small streams, blind channels of rivers, etc.), with substrate sandy or stony, water generally clear, and in most cases without vegetation. This species has a preference for exposed or semi-shaded situations (majority of observations in semi-forested environments).

#### ﻿ Pachynectes

Taxon classificationAnimaliaColeopteraDytiscidae

sp. Ma4

700E788A-891A-58FE-A743-455EE5FF89D3

##### Material examined.

1 ♂: MAK-5A; 2 ♂♂: MAK-5D; 1 ♂, 1 ♂: MAK-14A; 1 ♂, 1 ♀: MAK-29; 1 ♀: MAK-45; 8 ♂♂, 4 ♀♀: MAK-50; 1 ♂, 4 ♀♀: MAK-51; 57 ♂♂, 37 ♀♀: MAK-52; 1 ♀: MAK-59C.

##### Distribution.

Madagascar. So far endemic to the Makay massif.

##### Note.

This is an undescribed species, rather large for the genus, and very close to another undescribed species which lives in the Isalo massif.

##### Habitat in study area

**(Fig. 2F, N)**. Similar to *Pachynectes* sp. Ma1 (with which it was syntopic at all sites), but this species is rarer.

#### ﻿ Pseuduvarus

Taxon classificationAnimaliaColeopteraDytiscidae

sp. Ma1

F1EFAE25-10DA-5DBE-9805-2370F480F8E5

##### Material examined.

1 ♀: MAK-2; 1 ♂: MAK-61.

##### Distribution.

Madagascar (widespread).

##### Note.

This species corresponds to *P.ornatipennis* (Régimbart, 1900), currently wrongly considered a junior synonym of *P.vitticollis* (Boheman, 1848). A revision of the species of *Hydroglyphus* / *Pseuduvarus* is currently in preparation (J. Bergsten, pers. comm.).

##### Habitat in study area.

Same as *Clypeodytesconcivis* (see above).

#### ﻿ Uvarus betsimisarakus

Taxon classificationAnimaliaColeopteraDytiscidae

(Guignot, 1939)

745802C4-FD21-5371-98D1-E66838797117

##### Type locality.

Madagascar, Maroantsetra.

##### Material examined.

1 ♂, 1 ♀: MAK-19.

##### Distribution.

Madagascar (Guignot 1959–1961; Biström 1988c). Common in the Central Highlands.

##### Habitat in study area.

This species was collected only once, in a peripheral site located in a semi-open area impacted by human activities. The habitat was a large puddle partially sheltered by trees, with water slowly flowing and with abundant rice straw debris, on a dirty road between two rice fields.

#### ﻿ Uvarus rivulorum

Taxon classificationAnimaliaColeopteraDytiscidae

(Régimbart, 1895)

FC43306B-09DC-5932-A4F8-6050F214CBD2

= U.cilunculus Guignot, 1950.

##### Type locality.

Madagascar, Antsiranana.

##### Material examined.

1 ♀: MAK-1A; 2 ♀♀: MAK-2; 1 ♀: MAK-18; 4 ♂♂, 2 ♀♀: MAK-19; 4 exs.: MAK-60.

##### Distribution.

Madagascar, widespread in lowlands (Guignot 1959–1961; Rocchi 1991; Biström 1988c).

##### Habitat in study area

**(Fig. 2B, C).** This species was collected mainly at peripheral sites, in shallow lentic or slowly flowing lotic habitats, with or without marginal vegetation. The environment was open (non-forested), at some sites partly sheltered by sparse trees, and at most sites impacted by anthropogenic pressures (notably cattle trampling). The bottom varied from clay to sand and more or less abundant plant debris; the water was clear to turbid.

#### ﻿ Yola costipennis

Taxon classificationAnimaliaColeopteraDytiscidae

(Fairmaire, 1869)

B4643799-AA5F-5FC1-8A98-F9952F0ABB25

##### Type locality.

Madagascar, Sainte Marie Island.

##### Material examined.

5 exs.: MAK-1A; 2 exs.: MAK-1C; 4 exs.: MAK-19; 6 exs.: MAK-20; 4 exs.: MAK-41; 1 ex.: MAK-42; 1 ex.: MAK-60; 3 exs.: MAK-62.

##### Distribution.

Madagascar, widespread and common (Guignot 1959–1961; Bertrand and Legros 1971; Biström 1983; Bameul 1984; Rocchi 1991).

##### Habitat in study area

**(Fig. 2A).** This species was collected mainly at peripheral sites and always in open areas, often impacted by human activities. The habitats were various kinds of shallow lentic water bodies, most without but some with water circulation. They were sun-exposed (at one site partly sheltered by trees), with bottom of sand, clay, or a mix of sand and clay, with organic debris varying from absent to forming a thick layer above the mineral substratum, with water clear to moderately turbid; vegetation was absent or sparse, and filamentous green algae were either undetectable or variously developed.

###### Tribe Hydrovatini

#### ﻿ Hydrovatus acuminatus

Taxon classificationAnimaliaColeopteraDytiscidae

Motschulsky, 1860

12E8DC35-C5C4-5645-99B8-0B9EA73E755B

= H.affinis Régimbart, 1895; H.badius (Clark, 1863); H.consanguineus Régimbart, 1880; H.ferrugineus Zimmermann, 1919; H.humilis Sharp, 1882; H.malaccae (Clark, 1863); H.obscurus Motschulsky, 1860; H.obscurus Régimbart, 1895; H.sordidus Sharp, 1882.

##### Type locality.

South-East Asia (Indian continent).

##### Material examined.

1 ♂, 1 ♀: MAK-1A; 6 ♂♂, 6 ♀♀: MAK-2; 1 ♀: MAK-3; 2 ♂♂, 1 ♀: MAK-19; 1 ♂, 2 ♀♀: MAK-21; 1 ♂: MAK-23; 1 ♀: MAK-44C; 1 ♂, 2 ♀♀: MAK-60; 1 ♂, 1 ♀: MAK-61; 1 ♂: MAK-62.

##### Distribution.

Sub-Saharan Africa, Madagascar, Seychelles, Turkey, Egypt, Arabian Peninsula, south-eastern Palearctic region from India to south Japan, Oriental region (Biström 1996; Hájek and Reiter 2014). In Madagascar, widespread and common in lowlands (absent from the Central Highlands).

##### Habitat in study area

**(Fig. 2B–D).** This species is present in lentic and in slowly flowing lotic habitats. It was collected both at peripheral and inner massif sites. The bottom varied from clayey to sandy, with clear, red-brown or turbid water and with more or less abundant plant debris. This species has a clear preference for open environments and habitats with at least some vegetation and is highly tolerant to anthropogenic disturbance.

#### ﻿ Hydrovatus capnius

Taxon classificationAnimaliaColeopteraDytiscidae

Guignot, 1950

F3DA6FBE-C9A6-5B98-AADE-C3FF17226D44

##### Type locality.

Zambia (Congo Belge), Musosa.

##### Material examined.

1 ♂: MAK-2; 1 ♂, 1 ♀: MAK-42.

##### Distribution.

Sudan, Democratic Republic of the Congo, Madagascar (Guignot 1959–1961; Biström 1996). Distribution within Madagascar poorly known, but probably restricted to lowlands.

##### Habitat in study area

**(Fig. 2B, C).** This species was found at only two peripheral sites, in open and non-forested environments, both markedly eutrophic. The first site was a shallow isolated stream characterised by very weak water flow, sandy bottom, marked anthropic disturbance (cattle trampling), sparse tufts of small Cyperaceae and strong presence of filamentous green algae. The second site was an open marsh, relatively preserved from anthropogenic pressure, without water flow, with sandy bottom and moderate quantity of plant debris, moderately turbid water, sparse helophytes (*Cyperus*) and aquatic plants (*Nymphaea* and *Polygonum*), floating ferns (*Azolla*, an indicator of eutrophication) and moderate abundance of filamentous green algae.

#### ﻿ Hydrovatus crassicornis

Taxon classificationAnimaliaColeopteraDytiscidae

(H. J. Kolbe, 1883)

215BFFDA-F4E6-5F2B-BDF7-1CF5EA762FCE

##### Type locality.

Eastern part of Madagascar

##### Material examined.

1 ♂, 1 ♀: MAK-42; 1 ♂: MAK-60; 1 ♂, 1 ♀: MAK-61.

##### Distribution.

Madagascar, widespread (Guignot 1959–1961; Bertrand and Legros 1971; Biström 1996).

##### Habitat in study area.

This species was collected at two peripheral and one inner massif sites. The habitats were located in open areas and were lentic water bodies (with or without slow water circulation) with sand-clay bottom and with plant debris, with water clear to moderately turbid, with discontinuous marginal belts of helophytes and at one site with filamentous green algae.

#### ﻿ Hydrovatus cruentatus

Taxon classificationAnimaliaColeopteraDytiscidae

H. J. Kolbe, 1883

72C4C675-10EF-588C-A29B-C023ADBD671D

##### Type locality.

Eastern part of Madagascar.

##### Material examined.

1 ♂: MAK-2; 1 ♀: MAK-42.

##### Distribution.

Madagascar, widespread (Guignot 1959–1961; Biström 1996).

##### Habitat in study area.

Same as *Hydrovatuscapnius* (see above).

#### ﻿ Hydrovatus dentatus

Taxon classificationAnimaliaColeopteraDytiscidae

Bilardo & Rocchi, 1990

B33BB396-11B0-5624-A29C-7753FCC7D33C

##### Type locality.

Zambia, Luangwa valley, Chibembe.

##### Material examined.

1 ♂: MAK-2.

##### Distribution.

Zambia, South Africa (Biström 1996), Madagascar. For Madagascar, the first record is recent (Ranarilalatiana 2019) and was from Anjozorobe-Angavo in the Central Highlands.

##### Habitat in study area

**(Fig. 2B, C).** This species was sampled only once in a peripheral site. The habitat was a shallow insolated stream characterised by very weak water flow, sandy bottom, marked anthropic disturbance (cattle trampling), sparse tufts of small Cyperaceae and strong presence of filamentous green algae.

#### ﻿ Hydrovatus otiosus

Taxon classificationAnimaliaColeopteraDytiscidae

Guignot, 1945

407D478E-9662-5322-B13A-FE3CCA3CCD16

##### Type locality.

Madagascar, Antananarivo, Ikopa River.

##### Material examined.

3 ♂♂, 2 ♀♀: MAK-2; 3 ♂♂, 1 ♀: MAK-62.

##### Distribution.

Madagascar, widespread (Guignot 1959–1961; Bertrand and Legros 1971; Biström 1996); common in the Central Highlands.

##### Habitat in study area

**(Fig. 2B, C).** This species was collected at only two peripheral sampling sites. The first site was the one described above for *H.dentatus*. The second one was a canal at the edge of rice fields, with water slowly flowing, muddy bottom, water rather turbid, and without vegetation. Both sites were significantly impacted by anthropogenic disturbance.

#### ﻿ Hydrovatus parvulus

Taxon classificationAnimaliaColeopteraDytiscidae

Régimbart, 1900

6B4DE427-4F28-58D1-9DBC-5790C5A7CC56

= H.noctivagus Guignot, 1953; H.ocnerus Guignot, 1958; H.socors Guignot, 1954.

##### Type locality.

Madagascar, Antongil Bay.

##### Material examined.

2 ♂♂: MAK-2; 2 exs.: MAK-43; 3 exs.: MAK-61.

##### Distribution.

Sub-Saharan Africa, Madagascar (Guignot 1959–1961; Biström 1996). In Madagascar, widespread and common.

##### Habitat in study area

**(Fig. 2B, C).** This species was collected at three peripheral sampling sites, in open, non-forested environments. Two of the sites were lotic habitats (a shallow stream and the calm margin of a river) and one was lentic (a shallow pond ~ 25 m in diameter). In all cases, the bottom consisted of sand and clay, the water was clear, and the marginal zone where the beetles were collected was vegetated.

#### ﻿ Hydrovatus pictulus

Taxon classificationAnimaliaColeopteraDytiscidae

Sharp, 1882

517CA236-4733-5711-9469-7E15FD622F23

H. dilutus H. J. Kolbe, 1883; H.scymnoides Régimbart, 1895.

##### Type locality.

Madagascar.

##### Material examined.

5 ♂♂, 1 ♀: MAK-2; 1 ♂: MAK-62.

##### Distribution.

Sub-Saharan Africa, Madagascar (Guignot 1959–1961; Biström 1996). In Madagascar, widespread.

##### Habitat in study area.

Same as *Hydrovatusotiosus* (see above). Throughout Madagascar, this species has an ecological optimum in the calm parts of rivers and streams or their satellite puddles and pools, with sandy or muddy bottom and few or no vegetation and is quite tolerant to anthropogenic disturbance.

#### ﻿ Hydrovatus testudinarius

Taxon classificationAnimaliaColeopteraDytiscidae

Régimbart, 1895

EDDE447E-9595-57E2-AF35-61562E242B6E

##### Type locality.

Madagascar, Antananarivo, Ambodinandohalo Lake.

##### Material examined.

7 ♂♂, 3 ♀♀: MAK-2.

##### Distribution.

Madagascar, widespread but not common (Guignot 1959–1961; Rocchi 1991; Biström 1996).

##### Habitat in study area.

Same as *Hydrovatusdentatus* (see above).

#### ﻿ Hydrovatus

Taxon classificationAnimaliaColeopteraDytiscidae

sp. Ma7

FD7EFCAD-C66E-5335-BE13-072CFCD77A0E

##### Material examined.

1 ♂, 1 ♀: MAK-2.

##### Note.

This large species may be *H.confusus* Régimbart, 1903, a species endemic to Madagascar, or *H.badeni* Sharp, 1882, also present in continental Sub-Saharan Africa (Biström 1996).

##### Distribution.

Unknown.

##### Habitat in study area.

Same as *Hydrovatusdentatus* (see above)

###### Tribe Hyphydrini

#### ﻿ Hyphydrus separandus

Taxon classificationAnimaliaColeopteraDytiscidae

Régimbart, 1895

43165BFE-DA9F-5F5B-8A68-C0607CB0F45D

= H.oncodes Guignot, 1955.

##### Type locality.

Madagascar, Montagne d’Ambre, Ambohitra National Park.

##### Material examined.

2 ♂♂, 8 ♀♀: MAK-3; 2 ♀♀: MAK-4; 30 exs.: MAK-5A; 8 exs.: MAK-5B; 15 exs.: MAK-5C; 85 exs.: MAK-5D; 5 ♂♂, 6 ♀♀: MAK-8; 1 ♀: MAK-10; 1 ♀: MAK-11B; 3 ♂♂, 5 ♀♀: MAK-14A; 1 ♀: MAK-16; 1 ♂, 1 ♀: MAK-25B; 4 exs.: MAK-28; 14 exs.: MAK-29; 1 ♂: MAK-35B; 1 ♂, 1 ♀: MAK-37A; 5 ♂♂: MAK-40A; 1 ex.: MAK-40B; 1 ♂: MAK-50; 2 ♂♂, 1 ♀: MAK-52; 1 ♂: MAK-59C.

##### Distribution.

Comoro Islands, Madagascar (Guignot 1959–1961; Wewalka 1980; Biström 1982). In Madagascar, widespread.

##### Habitat in study area

**(Fig. 2D–F, I, L–N, P).** This species was collected only in inner Makay where it is one of the most common species of aquatic Adephaga. It was found in puddles, pools, ponds and slowly flowing streams, from a few centimetres to 150 cm in depth, with sandy or rocky bottom, clear water and more or less abundant plant debris, often without vegetation. Almost all collecting sites were located in forested or semi-forested environment.

#### ﻿ Hyphydrus stipes

Taxon classificationAnimaliaColeopteraDytiscidae

Sharp, 1882

BD3164EB-861C-58FC-B91F-40901497BCD4

= H.soarezicus Alluaud, 1897.

##### Type locality.

Madagascar.

##### Material examined.

1 ♂: MAK-29.

##### Distribution.

Madagascar, widespread (Biström 1982; Rocchi 1991).

##### Habitat in study area.

This species was collected at a single site located in inner Makay, in a semi-forested small valley. The habitat was a sun-exposed isolated pool on sandstone rock, rather deep (70 cm), with bottom made up of sand and stones with a moderate quantity of plant debris, with clear water and no vegetation. This site was well preserved from anthropogenic disturbance.

###### Tribe Methlini

#### ﻿ Methles

Taxon classificationAnimaliaColeopteraDytiscidae

sp. Ma1

6AD22F63-6B67-5FD1-8A99-34F1817CA2AB

##### Material examined.

4 ♂♂, 3 ♀♀: MAK-44C; 1 ♂, 2 ♀♀: MAK-60.

##### Note.

The Malagasy species of the genus *Methles* are in great need of revision, and in the current state of knowledge we cannot assign a name to this species.

##### Distribution.

Unknown (but not confined to the Makay area).

##### Habitat in study area.

This species was collected at two inner massif sites in northern Makay, with very different habitat characteristics. One was a blind channel in forest connected to River Sakapaly; this site is the locus typicus of *Copelatusmalavergnorum* sp. nov. (see “Habitat” under description of this species). The other site was an open marsh with vegetated margins (Cyperaceae and Polygonaceae), with muddy bottom and water rather turbid, in semi-forested context near the Sakapaly River. Both sites were preserved from anthropogenic disturbance.

#### ﻿ Methles

Taxon classificationAnimaliaColeopteraDytiscidae

sp. Ma5

2F4450B0-1EC0-5F89-9F7F-FFC21CD15C29

##### Material examined.

1 ♀: MAK-2; 3 ♂♂, 3 ♀♀: MAK-42; 1 ♀: MAK-43; 1 ♂: MAK-44C; 1 ♂, 3 ♀♀: MAK-60; 19 ♂♂, 24 ♀♀: MAK-61; 3 ♂♂, 1 ♀: MAK-62.

##### Note.

The Malagasy species of the genus *Methles* are in great need of revision, and in the current state of knowledge we cannot assign a name to this species.

##### Distribution.

Unknown (but not confined to the Makay area).

##### Habitat in study area

**(Fig. 2B, C).** This species is present in lentic and in slowly flowing lotic habitats. It was collected both at peripheral and inner massif sites. The bottom varied from clay to sandy, with more or less abundant plant debris. The water was either clear, red-brown or turbid. This species has a clear preference for open environments and habitats with at least some vegetation and is tolerant to anthropogenic disturbance.

###### Subfamily Laccophilinae, tribe Laccophilini

#### ﻿ Africophilus bartolozzii

Taxon classificationAnimaliaColeopteraDytiscidae

Rocchi, 1991

11A6DFB8-A6C4-5F52-A2C8-AD8CBF440CFF

##### Type locality.

Madagascar, Isalo National Park, Canyon des Singes.

##### Material examined.

1 ♀: MAK-8; 8 ♂♂, 8 ♀♀: MAK-9; 1 ♀: MAK-36B; 1 ♀: MAK-39B.

##### Distribution.

Madagascar; previously known only from the sandstone massif of Isalo (Rocchi 1991).

##### Habitat in study area

**(Fig. 2H, I).** This hygropetric species was collected only in inner Makay. Three of the sampling sites were situated in forested contexts and one site in a non-forested context. In the latter (MAK-9), the habitat was vertical rock walls with water film and crust of bryophytes and algae, at the bottom of a narrow and deep canyon. The other habitats were puddles and pools with sandy – stony bottom along small streams. Like other members of the genus, this species lives at the edge of small water bodies in the water film retained by capillarity on the substratum surface above water line, or on rock surface covered with a thin and slowly seeping water film, often close to cascades.

#### ﻿ Africophilus nesiotes

Taxon classificationAnimaliaColeopteraDytiscidae

Guignot, 1951

5D89B900-B7DA-58F4-8AAA-8EBA3B77BA90

##### Type locality.

Madagascar, Ambalavao region.

##### Material examined.

5 ♂♂, 8 ♀♀: MAK-5A; 1 ♀: MAK-5B; 1 ♀: MAK-8; 2 ♀♀: MAK-13; 3 ♂♂, 4 ♀♀: MAK-14A; 1 ♀: MAK-26; 1 ♀: MAK-39A; 2 ♂♂: MAK-40A

##### Distribution.

Sub-Saharan Africa, Madagascar (Guignot 1959–1961; Bertrand and Legros 1971; Franciscolo 1994; Bilardo et al. 2020). In Madagascar, widespread.

##### Habitat in study area

**(Fig. 2F, I, N).** Similar to *A.bartolozzii* (see above) but more common.

#### ﻿ Laccophilus addendus

Taxon classificationAnimaliaColeopteraDytiscidae

Sharp, 1882

BCD071B2-A28E-5785-AC0B-73522639A168

##### Type locality.

Madagascar.

##### Material examined.

1 ♀: MAK-2; 1 ♀: MAK-5A; 28 ♂♂, 19 ♀♀: MAK-11A; 1 ♀: MAK-11B; 2 ♂♂, 1 ♀: MAK-19; 2 ♂♂, 1 ♀: MAK-25A; 1 ♂: MAK-26; 1 ♂: MAK-38A; 1 ♂, 1 ♀: MAK-62.

##### Distribution.

Madagascar, widespread (Guignot 1959–1961; Bameul 1984; Rocchi 1991; Biström et al. 2015). Previous records from outside Madagascar are considered uncertain by Biström et al. (2015).

##### Habitat in study area

**(Fig. 2B, C, F, O, P).** This species was collected at peripheral and inner massif sites, mainly in non-forested areas, in lentic or very slow flowing lotic habitats, most often sun-exposed but at a few sites partly shaded by trees. The bottom consisted of various amounts of sand, clay and mud, with moderate to abundant plant debris, and marginal vegetation was absent to well developed. The water was clear to rather turbid. This species occurs both in strongly anthropised contexts and in habitats preserved from anthropogenic pressure.

#### ﻿ Laccophilus flaveolus

Taxon classificationAnimaliaColeopteraDytiscidae

Régimbart, 1906

D6B6AE2B-BC6C-5DBF-A2D2-801DBFB1F78B

= L.pampinatus Guignot, 1941.

##### Type locality.

Kenya, Winam, Kavirondo Bay.

##### Material examined.

1 ♂: MAK-2.

##### Distribution.

Eastern Sub-Saharan Africa, Madagascar (Guignot 1959–1961; Bameul 1984; Biström et al. 2015).

##### Habitat in study area

**(Fig. 2B, C).** This species was sampled only once at a peripheral site. The habitat was a shallow insolated stream characterised by very weak water flow, sandy bottom, marked anthropic disturbance (cattle trampling), sparse tufts of small Cyperaceae and strong presence of filamentous green algae.

#### ﻿ Laccophilus insularum

Taxon classificationAnimaliaColeopteraDytiscidae

Biström, Nilsson & Bergsten, 2015

59F6BE32-D147-525C-8F99-CE88AFD08254

##### Type locality.

Madagascar, Ankarafantsika National Park, Mahajanga, Boeny.

##### Material examined.

2 ♂♂, 2 ♀♀: MAK-5A.

##### Distribution.

Madagascar, widespread (Biström et al. 2015).

##### Habitat in study area

**(Fig. 2F).** This species was encountered only once, in south-central inner Makay. The habitat was a deep pond (dimensions about 30 m × 15 m) at the bottom of a small canyon along the course of a stream. This water body was formed as a result of the collapse of downstream canyon walls (natural dam). It was shaded by the canyon walls and surrounded by trees (notably *Cyathea* tree ferns) and hygrophilous vegetation. The habitat was furthermore characterised by clay bottom, with moderate amount of plant debris, clear water, and absence of marginal vegetation. This site was unaffected by anthropic disturbance.

#### ﻿ Laccophilus luctuosus

Taxon classificationAnimaliaColeopteraDytiscidae

Sharp, 1882

F7DAADE9-41DF-5F1B-B5C3-F2EC89A8DC6A

##### Type locality.

Madagascar.

##### Material examined.

1 ♂, 1 ♀: MAK-21.

##### Distribution.

Madagascar (widespread in lowlands) (Guignot 1959–1961; Bertrand and Legros 1971; Rocchi 1991; Biström et al. 2015).

##### Habitat in study area.

This species was encountered only once in a peripheral site located in an open area. The habitat was a small isolated temporary puddle with sandy bottom and clear water under *Phragmites*, on the west bank of the Makaikely River. There was a moderate amount of plant debris, and no vegetation.

#### ﻿ Laccophilus makay

Taxon classificationAnimaliaColeopteraDytiscidae

Manuel & Ramahandrison, 2020

ACDD693E-6E1B-50E3-BE82-FCB09EECE56C

##### Type locality.

Madagascar, Toliara, Makay massif, 10.7 km NW of Tsivoky.

##### Material examined.

8 ♂♂, 7 ♀♀: MAK-3; 2 ♂♂, 1 ♀: MAK-4; 4 ♂♂, 1 ♀: MAK-5A; 3 ♂♂: MAK-5B; 2 ♂♂: MAK-5C; 7 ♂♂, 5 ♀♀: MAK-5D; 1 ♂, 2 ♀♀: MAK-6; 1 ♂, 1 ♀: MAK-7; 11 ♂♂, 9 ♀♀: MAK-8; 2 ♂♂, 4 ♀♀: MAK-10; 13 ♂♂, 6 ♀♀: MAK-14A; 3 ♂♂, 2 ♀♀: MAK-15; 1 ♂, 1 ♀: MAK-16; 1 ♂, 1 ♀: MAK-25A; 9 ♂♂, 22 ♀♀: MAK-25B; 1 ♂, 1 ♀: MAK-26; 4 ♂♂, 4 ♀♀: MAK-28; 1 ♀: MAK-29; 5 ♂♂, 2 ♀♀: MAK-30; 3 ♂♂, 5 ♀♀: MAK-31A; 5 ♂♂, 2 ♀♀: MAK-31B; 4 ♂♂, 1 ♀: MAK-31C; 12 ♂♂, 20 ♀♀: MAK-32; 1 ♂, 4 ♀♀: MAK-33; 1 ♂: MAK-34A; 2 ♂♂, 3 ♀♀: MAK-34B; 11 ♂♂, 3 ♀♀: MAK-35A; 4 ♂♂: MAK-35B; 5 ♂♂: MAK-35C; 6 ♂♂, 5 ♀♀: MAK-36B; 8 ♂♂, 11 ♀♀: MAK-38A; 1 ♂, 1 ♀: MAK-39A; 3 ♂♂, 1 ♀: MAK-39B; 1 ♂: MAK-44C; 68 exs.: MAK-45; 2 ♂♂, 5 ♀♀: MAK-46; 4 ♂♂, 6 ♀♀: MAK-47; 21 exs.: MAK-49; 130 exs.: MAK-50; 26 exs.: MAK-51; 111 exs.: MAK-52; 108 exs.: MAK-53; 16 ♂♂, 19 ♀♀: MAK-54A; 64 exs: MAK-54B; 8 ♂♂, 1 ♀: MAK-58; 37 exs.: MAK-59A; 5 ♂♂, 2 ♀♀: MAK-59B; 62 exs: MAK-59C.

##### Distribution.

So far endemic to the Makay massif, Madagascar (Manuel and Ramahandrison 2020).

##### Habitat in study area

**(Fig. 2D–G, I–N).** This species was collected only at inner massif sites, in both south-central and northern Makay, where it is by far the most common and abundant species of aquatic Adephaga. It was found in a wide diversity of lentic water bodies (puddles, pools, ponds, a blind river channel, etc.), isolated or with slow water renewal, as well as in very slowly flowing streams. The surrounding environment was forested or semi-forested and free from anthropisation. These habitats were further characterised by sandy bottom (sometimes with stones), various amounts of plant debris, clear water (but often with orange masses of iron bacteria), and marginal vegetation absent or poorly developed.

#### ﻿ Laccophilus pallescens

Taxon classificationAnimaliaColeopteraDytiscidae

Régimbart, 1903

68D7D120-8FFE-5424-92C1-10A0E5A11522

##### Type locality.

Southern Madagascar, Pays Androy.

##### Material examined.

1 ♀: MAK-1A; 8 ♂♂, 5 ♀♀: MAK-2; 1 ♂: MAK-18; 1 ♂: MAK-19.

##### Distribution.

Sub Saharan-Africa, Arabian Peninsula, Madagascar (Guignot 1959–1961; Bertrand and Legros 1971; Hájek and Reiter 2014; Biström et al. 2015).

##### Habitat in study area

**(Fig. 2B, C).** This species was collected only at peripheral sites, in shallow lentic or slowly flowing lotic habitats, with or without marginal vegetation. The environment was open (non-forested), at some sites partly sheltered by sparse trees, and at most sites impacted by anthropogenic pressures (notably cattle trampling). The bottom varied from clay to sand and more or less abundant plant debris, and the water was clear to turbid.

#### ﻿ Laccophilus posticus

Taxon classificationAnimaliaColeopteraDytiscidae

Aubé, 1838

63E8D9F3-467B-5892-B706-06E7B345E09E

##### Type locality.

Mascarene Islands, Mauritius.

##### Material examined.

5 ♂♂: MAK-1A; 1 ♂: MAK-2; 1 ♀: MAK-3; 3 ♀♀: MAK-4; 4 ♂♂, 3 ♀♀: MAK-11A; 1 ♂: MAK-11B; 1 ♂: MAK-17; 1 ♂: MAK-18; 243 exs.: MAK-19; 1 ♀: MAK-21; 3 ♂♂, 9 ♀♀: MAK-23; 1 ♀: MAK-28; 1 ♂, 2 ♀♀: MAK-40A; 11 ♂♂, 14 ♀♀: MAK-41; 37 exs.: MAK-42; 1 ♂: MAK-44A; 1 ♂: MAK-59C; 4 ♂♂, 6 ♀♀: MAK-60; 6 ♂♂, 1 ♀: MAK-62.

##### Distribution.

Mauritius, Aldabra, Madagascar (Guignot 1959–1961; Bertrand and Legros 1971; Bameul 1984; Rocchi 1991; Biström et al. 2015). In Madagascar, widespread and common in lowlands.

##### Habitat in study area

**(Fig. 2B–E, O, P).** This species was captured both at peripheral and inner massif sites, in various kinds of lentic habitats (puddles, pools, ponds, ditches) and in very slowly flowing streams. These habitats were most often located in open areas. The bottom variously consisted of sand, clay or stones, generally with plant debris. The water was clear to turbid and marginal vegetation was absent or variously developed. This species is highly tolerant to anthropogenic pressure.

#### ﻿ Laccophilus rivulosus

Taxon classificationAnimaliaColeopteraDytiscidae

Klug, 1833

AAA17401-1790-5ED3-9A0A-C36F4C128034

##### Type locality.

Madagascar.

##### Material examined.

1 ♀: MAK-2; 2 ♂♂, 7 ♀♀: MAK-19.

##### Distribution.

Madagascar, widespread but not very common (Guignot 1959–1961; Bertrand and Legros 1971; Biström et al. 2015).

##### Habitat in study area

**(Fig. 2B, C).** This species was found at two sampling sites located in the periphery of the Makay massif: a shallow isolated stream characterised by very weak water flow, sandy bottom, sparse tufts of small Cyperaceae and strong presence of filamentous green algae, and a large puddle partially sheltered by trees, with water slowly flowing and with abundant rice straw debris, on a dirty road between two rice fields. These habitats were situated in open environments and were impacted by anthropogenic disturbance (notably cattle trampling).

#### ﻿ Laccophilus seyrigi

Taxon classificationAnimaliaColeopteraDytiscidae

Guignot, 1937

74F6D2CB-69B6-5E91-9CC0-6727252E4ADD

##### Type locality.

Southern Madagascar, near Bekily.

##### Material examined.

1 ♀: MAK-2.

##### Distribution.

South and south-western Madagascar (very rare); the present record is the first since the original description of the species (Guignot 1959–1961; Biström et al. 2015).

##### Habitat in study area.

Same as *Laccophilusflaveolus* (see above).

#### ﻿ Laccophilus transversovittatus

Taxon classificationAnimaliaColeopteraDytiscidae

Biström, Nilsson & Bergsten, 2015

916F2599-E902-5AA5-AD6F-CBF320792047

##### Type locality.

Madagascar, Isalo, Menamaty River.

##### Material examined.

1 ♂, 1 ♀: MAK-12C; 1 ♀: MAK-26; 1 ♀: MAK-52.

##### Distribution.

Madagascar, widespread outside from the Central Highlands (Biström et al. 2015). The record from Ankaratra in Biström et al. (2015) is probably attributable to *L.rakouthae* Manuel & Ramahandrison, 2020.

##### Habitat in study area.

This species was found at three inner massif sites, two in south-central and one in northern Makay. The surrounding was forested or semi-forested and without visible anthropogenic disturbance. The habitats were: a small pond with water slowly renewed, just downstream from a spring, partly sheltered by trees, with clay bottom, with plant debris and clear water, with sparse helophytes (*Cyperus*); a slowly flowing stream, partly shaded, with sandy bottom, important accumulation of plant debris, clear water and sparse helophytes; and a small, isolated pool, partly shaded, with sandy bottom, no plant debris, tinted water, and no marginal vegetation.

#### ﻿ Laccophilus

Taxon classificationAnimaliaColeopteraDytiscidae

sp. Ma19

B9211C72-1C09-542E-BEE1-492B7B1A498D

##### Material examined.

1 ♂: MAK-21.

##### Note.

This is an undescribed species close to *L.lateralis* Sharp, 1882.

##### Distribution.

Madagascar (widespread in lowlands).

##### Habitat in study area.

Same as *Laccophilusluctuosus* (see above).

#### ﻿ Neptosternus oblongus

Taxon classificationAnimaliaColeopteraDytiscidae

Régimbart, 1895

C9D8363F-971F-52FF-BF0E-72324000CF28

##### Type locality.

Madagascar, Annanarivo (= Antananarivo?).

##### Material examined.

35 ♂♂, 50 ♀♀: MAK-5A; 3 ♂♂: MAK-5B.

##### Distribution.

Central and southern Madagascar (Guignot 1959–1961; Bilardo and Rocchi 2012). Distribution within the island poorly known.

##### Habitat in study area.

Same as *Laccophilusinsularum* (see above).

#### ﻿ Philaccolus elongatus

Taxon classificationAnimaliaColeopteraDytiscidae

(Régimbart, 1903)

2EA0903F-3C6E-5956-95CA-7080D971E29B

##### Type locality.

Madagascar, Sainte Marie Island.

##### Material examined.

1 ♂: MAK-2.

##### Note.

There is a complex of very similar species around *P.elongatus*, and future studies may show that the specimen recorded here belong to a different species.

##### Distribution.

Madagascar (Guignot 1959–1961; Bertrand and Legros 1971). Distribution within the island poorly known.

##### Habitat in study area.

Same as *Laccophilusflaveolus* (see above).

### ﻿Comparisons of species frequency, diversity, and endemism in different areas and vegetation contexts

Relative frequencies of occurrence of species across samplings for different sets of sampling sites (all, inner Makay, peripheral Makay, forested sites, semi-forested sites, non-forested sites) are given in Table 1. With samplings performed in the peripheral plain surrounding the Makay massif, the most dominant species (RFO > 20%; following species list ranked according to RFO value) were *Laccophilusposticus* (RFO 64.4%; all following species with RFO ≤ 50%), *Canthydrusguttula*, *Hydrovatusacuminatus*, *Yolacostipennis*, *Neohydrocoptusseriatus*, *Hydaticusservillianus*, *Cybistercinctus*, *Bidessusperexiguus*, *Hydroglyphusgeminodes*, *Bidessuslongistriga*, *Pachynectescostulifer*, *Methles* sp. Ma5, *Uvarusrivulorum*, *Laccophiluspallescens*, *L.addendus*, *Hydaticusdorsiger*, and *Rhantaticuscongestus*. For samplings in inner Makay, the most dominant species (same criteron and listing order) were *Laccophilusmakay* (RFO 65.6%; all following species with RFO < 36%), *Pachynectes* sp. Ma1, *Copelatusruficapillus*, *Hyphydrusseparandus*, *Copelatusacamas*, and *Dineutusproximus*. Hence, with a RFO threshold of 20%, there is not a single species in common among dominant species of inner vs. peripheral Makay. When the threshold is lowered to a RFO of 10%, only *Laccophilusposticus* (RFO peripheral: 64.4%; inner: 13.7%) shows up among dominant species in both areas. The species community of aquatic Adephaga populating the freshwater biota associated with the sandstone canyons of inner Makay is therefore highly original with respect to the community associated with the surrounding plain, the latter reflecting what can be found virtually anywhere in the western lowlands of Madagascar, in water bodies located in more or less deforested and man-impacted environments.

**Table 1.** List of the species sampled, with indication of status and values of relative frequencies of occurrence (RFO). E: endemic of Madagascar; E*: endemic of the Malagasy region; W (widespread): distribution extending outside the Malagasy region. NbOc (third column): total number of occurrences, i.e., number of samplings in which the species was present out of the 87 samplings performed. For RFO calculated by categories of sites, total number of samplings for each category indicated between parentheses in column headings.

Species	Status	NbOc (RFO %) all samplings (n = 87)	RFO % inner Makay (n = 73)	RFO % peripheral Makay (n = 14)	RFO % forested sites (n = 35)	RFO % semi-forested sites (n = 34)	RFO % non-forested sites (n = 18)
**Gyrinidae**							
*Dineutusproximus*	E	15 (17.2)	20.6	0	28.6	14.7	0
*D.s.sinuosipennis*	E	8 (9.2)	11.0	0	14.3	8.8	0
*Orectogyrusvicinus*	E	5 (5.7)	6.9	0	11.4	2.9	0
**Haliplidae**							
*Peltodytesquadratus*	E	2 (2.3)	0	14.3	0	0	11.1
**Noteridae**							
*Canthydrusconcolor*	E	1 (1.1)	0	7.1	0	0	5.6
*C.flavosignatus*	W	2 (2.3)	0	14.3	0	0	11.1
*C.guttula*	E*	11 (12.6)	5.5	50.0	5.7	2.9	44.4
*C.* sp. Ma5	?	3 (3.4)	1.4	14.3	0	2.9	11.1
*Neohydrocoptusseriatus*	W	12 (13.8)	8.2	42.9	8.6	5.9	38.9
*N.* sp. Ma3	?	3 (3.4)	4.1	0	5.7	2.9	0
*Sternocanthusfabiennae*	E	2 (2.3)	0	14.3	0	0	11.1
*Synchortusasperatus*	E	2 (2.3)	0	14.3	0	0	11.1
**Dytiscidae**							
**Copelatinae**							
*Copelatusacamas*	E	20 (23.0)	27.4	0	25.7	29.4	5.6
*C.andobonicus*	E	3 (3.4)	4.1	0	5.7	2.9	0
*C.polystrigus*	W	12 (13.8)	16.4	0	20.0	8.8	11.1
*C.ruficapillus*	E	21 (24.1)	28.8	0	25.7	32.3	5.6
*C.vigintistriatus*	E*	4 (4.6)	5.5	0	8.6	2.9	0
*C.malavergnorum* sp. nov.	E	1 (1.1)	1.4	0	2.9	0	0
*C.zanabato* sp. nov.	E	2 (2.3)	2.7	0	0	5.9	0
*Madaglymbusfairmairei*	E	12 (13.8)	16.4	0	20.0	11.8	5.6
**Cybistrinae**							
*Cybistercinctus*	E	6 (6.9)	0	42.9	0	0	33.3
*C.operosus*	E	1 (1.1)	1.4	0	0	2.9	0
**Dytiscinae, Aciliini**							
*Rhantaticuscongestus*	W	3 (3.4)	0	21.4	0	0	16.7
**Dytiscinae, Eretini**							
*Eretesgriseus*	W	1 (1.1)	1.4	0	0	0	5.6
**Dytiscinae, Hydaticini**							
*Hydaticusdorsiger*	W	7 (8.0)	5.5	21.4	2.9	2.9	27.8
*H.exclamationis*	W	1 (1.1)	1.4	0	0	0	5.6
*H.petitii*	E	1 (1.1)	1.4	0	0	2.9	0
*H.servillianus*	W	7 (8.0)	1.4	42.9	2.9	0	33.3
*H.sobrinus*	E*	5 (5.7)	6.8	0	5.7	8.8	0
**Hydroporinae, Bidessini**							
*Bidessuslongistriga*	E	6 (6.9)	1.4	35.7	0	2.9	27.8
*B.perexiguus*	E	7 (8.0)	2.7	35.7	0	5.9	27.8
*Clypeodytesconcivis*	E	2 (2.3)	0	14.3	0	0	11.1
*C.insularis*	E	1 (1.1)	0	7.1	0	0	5.6
*C.* sp. Ma3	?	1 (1.1)	0	7.1	0	0	5.6
*Hydroglyphuscapitatus*	E*	3 (3.4)	1.4	7.1	0	2.9	5.6
*H.geminodes*	W	7 (8.0)	2.7	35.7	0	5.9	27.8
*H.plagiatus*	E	1 (1.1)	1.4	0	0	2.9	0
*Liodessusluteopictus*	E*	1 (1.1)	1.4	0	2.9	0	0
*Pachynectescostulifer*	E	5 (5.7)	0	35.7	0	0	27.8
*P.* sp. Ma1	E	27 (31.0)	35.6	7.1	31.4	44.1	5.6
*P.* sp. Ma4	E	9 (10.3)	12.3	0	5.7	20.6	0
*Pseuduvarus* sp. Ma1	?	2 (2.3)	0	14.3	0	0	11.1
*Uvarusbetsimisarakus*	E	1 (1.1)	0	7.1	0	0	5.6
*U.rivulorum*	E	5 (5.7)	1.4	28.6	0	2.9	22.2
*Yolacostipennis*	E	8 (9.2)	1.4	50.0	0	2.9	38.9
**Hydroporinae, Hydrovatini**							
*Hydrovatusacuminatus*	W	10 (11.5)	4.1	50.0	2.9	5.9	38.9
*H.capnius*	W	2 (2.3)	0	14.3	0	0	11.1
*H.crassicornis*	E	3 (3.4)	1.4	7.1	0	2.9	5.6
*H.cruentatus*	E	2 (2.3)	0	14.3	0	0	11.1
*H.dentatus*	W	1 (1.1)	0	7.1	0	0	5.6
*H.otiosus*	E	2 (2.3)	0	14.3	0	0	11.1
*H.parvulus*	W	3 (3.4)	1.4	7.1	0	2.9	5.6
*H.pictulus*	W	2 (2.3)	0	14.3	0	0	11.1
*H.testudinarius*	E	1 (1.1)	0	7.1	0	0	5.6
*H.* sp. Ma7	?	1 (1.1)	0	7.1	0	0	5.6
**Hydroporinae, Hyphydrini**							
*Hyphydrusseparandus*	E*	21 (24.1)	28.8	0	25.7	32.4	5.6
*H.stipes*	E	1 (1.1)	1.4	0	0	2.9	0
**Hydroporinae, Methlini**							
*Methles* sp. Ma1	?	2 (2.3)	2.7	0	2.9	2.9	0
*M.* sp. Ma5	?	7 (8.0)	4.1	28.6	2.9	5.9	22.2
**Laccophilinae**							
*Africophilusbartolozzii*	E	4 (4.6)	5.5	0	8.6	0	5.6
*A.nesiotes*	W	8 (9.2)	11.0	0	17.1	5.9	0
*Laccophilusaddendus*	E	9 (10.3)	8.2	21.4	5.7	5.9	27.8
*L.flaveolus*	W	1 (1.1)	0	7.1	0	0	5.6
*L.insularum*	E	1 (1.1)	1.4	0	0	2.9	0
*L.luctuosus*	E	1 (1.1)	0	7.1	0	0	5.6
*L.makay*	E	48 (55.2)	65.6	0	62.9	73.5	5.6
*L.pallescens*	W	4 (4.6)	0	28.6	0	0	22.2
*L.posticus*	E*	19 (21.8)	13.7	64.3	8.6	14.7	61.1
*L.rivulosus*	E	2 (2.3)	0	14.3	0	0	11.1
*L.seyrigi*	E	1 (1.1)	0	7.1	0	0	5.6
*L.transversovittatus*	E	3 (3.4)	4.1	0	2.9	5.9	0
*L.* sp. Ma19	E	1 (1.1)	0	7.1	0	0	5.6
*Neptosternusoblongus*	E	2 (2.3)	2.7	0	0	5.9	0
*Philaccoluselongatus*	E	1 (1.1)	0	7.1	0	0	5.6

According to vegetation contexts, dominant species (RFO > 20%) in forested areas were *Laccophilusmakay* (RFO 62.9%, all following species with RFO < 32%), *Pachynectes* sp. Ma1, *Dineutusproximus*, *Copelatusruficapillus*, *Hyphydrusseparandus*, *Copelatusacamas*, *C.polystrigus*, and *Madaglymbusfairmairei*. For semi-forested sites (for how “forested” vs. “semi-forested” environments were defined in this study, see Material and methods, Categories of sampling sites), species with RFO > 20% were *Laccophilusmakay* (RFO 73.5%; all following species with RFO < 45%), *Pachynectes* sp. Ma1, *Hyphydrusseparandus*, *Copelatusruficapillus*, *C.acamas*, and *Pachynectes* sp. Ma4. Thus, dominant species of aquatic Adephaga are largely the same in forested and semi-forested areas of inner Makay (these two environment categories not comprising any peripheral site), but with some differences. Some species seem to prefer water bodies located in forest (RFO “forested” vs. “semi-forested”; *Dineutusproximus*: 28.6% vs. 14.7%; *Madaglymbusfairmairei*: 20% vs. 11.8%). Another example of a species found predominantly in forested contexts (but with RFO < 20% in both) is *Orectogyrusvicinus* with 11.4% vs. 2.9%. In constrast, species of the genus *Pachynectes* seem to prefer semi-open contexts (*Pachynectes* sp. Ma1: 31.4% vs. 44.1%; *Pachynectes* sp. Ma4: 5.7% vs. 20.6%). The remaining dominant species in inner Makay sites have similar RFO values in forested vs. semi-forested contexts (e.g., *Laccophilusmakay*, *Copelatusruficapillus*, *C.acamas*, *Hyphydrusseparandus*; Table 1). Dominant species for non-forested sites are largely the same as those listed above for peripheral sites (most non-forested sites being located in the peripheral area), and therefore are completely different from the dominant species in forested and semi-forested sites.

**Figure 5.**
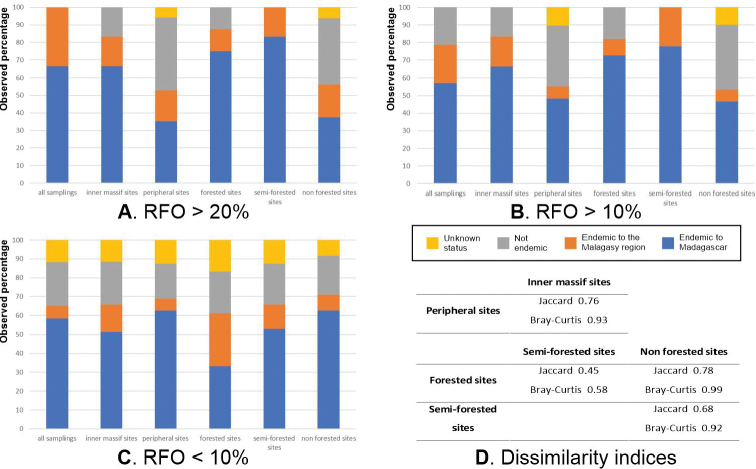
Comparison across site categories of percentages of endemics for dominant or rare species, and calculated dissimilarity indices for species composition **A–C** percentages of endemics among species whose relative frequency of occurrence (RFO), for the corresponding category of sites, is above or below a certain threshold **D** calculated values of Jaccard and Bray-Curtis dissimilarity indices between pairs of site categories.

We also computed dissimilarity indices based on all species to see how the whole community varies across categories of sites (Jaccard dissimilarity index, calculated from occurrence data; Bray-Curtis dissimilarity index, taking into account numbers of individuals captured for each species; Fig. 5D). This approach confirms that sites located in the peripheral plain vs. in inner Makay have very different species composition (Jaccard index: 0.76; Bray-Curtis index: 0.93; Fig. 5D). Sites in forested vs. semi-forested context support more similar communities (Jaccard index: 0.45; Bray-Curtis index: 0.58), whereas sites in non-forested environment harbour communities that are highly different both to those in forested environment (Jaccard index: 0.78; Bray-Curtis index: 0.99) and to a lesser extent in semi-forested environment (Jaccard index: 0.68; Bray-Curtis index: 0.92; Fig. 5D).

Observed percentages of species endemic to Madagascar are 58.1% for all samplings, 55.3% for inner Makay, 53.3% for peripheral Makay, 48.3% for forested sites, 58.5% for semi-forested sites and 53.7% for non-forested sites. Thus, when all sampled species are considered, rather surprisingly, endemicity is very similar in inner Makay vs. in the (deforested) peripheral plain and is also similar across categories of environment with the lowest value for forested areas, the highest for semi-forested areas, and non-forested areas standing in-between. When looking at percentages of endemic species among dominant species (RFO > 20% or RFO > 10%), a different picture emerges (Fig. 5A–C). Among species with RFO > 20% (Fig. 5A), the percentage of endemics is 83.3% for samplings in inner Makay and only 41.2% for samplings in the peripheral area. With a 10% cut-off (Fig. 5B), the pattern is similar although the magnitude of the difference is smaller. For vegetation contexts, endemicity for dominant species (RFO > 20%) is 75% in forested environment, 83.3% in semi-forested environment, and only 43.8% in non-forested environment (Fig. 5A); here again, lowering the cut-off to RFO 10% yields a similar pattern (Fig. 5B). When considering now only rare species (RFO < 10%) (Fig. 5C), for sites located in forest, the percentage of endemics is only 33.3%, whereas for non-forested sites, it is 62.5%. This opposite pattern of contrasted endemism levels for dominant vs. rare species is also apparent when comparing inner Makay sites (high endemism for dominant species, low endemism for rare species) and peripheral sites (vice-versa) (Fig. 5A–C). Indeed, a large fraction of the species that are found only occasionally in inner Makay are species that are extremely common in the peripheral area, and more generally in western Madagascar lowlands, most being non endemic (e.g., *Laccophilusposticus*, *Hydrovatusacuminatus*, *Hydroglyphusgeminodes*, *Hydaticusdorsiger*, *H.servillianus*, etc.).

**Figure 6.**
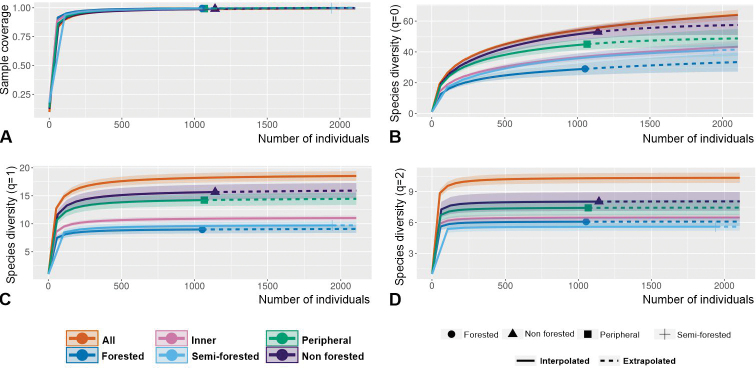
Interpolation-extrapolation graphs for the whole Makay dataset (All), for samplings in inner and in peripheral Makay, and for samplings in different vegetation contexts. Coloured lines represent the interpolated (solid line) or extrapolated (dashed line) estimate of the metric against number of individuals; the surface of lighter colour surrounding each curve materialises the 95% confidence interval **A** sample coverage **B** Hill number of order q=0 (H_0_ or species richness) **C** Hill number of order q = 1 (H_1_) **D** Hill number of order q = 2 (H_2_).

Observed species richness (number of species counted in the samplings) was 74 for all samplings, 45 for peripheral Makay, 47 for inner Makay, 29 for forested sites, 41 for semi-forested sites and 54 for deforested sites. Because observed species richness is a very poor proxy for species diversity, we ran interpolation-extrapolation analyses to obtain estimates of the H_0_, H_1_ and H_2_ metrics (see Material and methods) for the various categories of sites (Fig. 6). For all of these categories, sample coverage plotted against number of individuals attained a plateau well before reaching the extrapolated part of the curve, and was > 0.99 with the observed number of individuals (Fig. 6A). This means that the samples sufficiently cover the original communities for estimates of species diversity to be accurate. For species richness (H_0_), interpolation suggests that the number of species is higher in peripheral than in inner Makay. Indeed, a random sampling of 1000 individuals (this number being just below the minimal number of specimens sampled for any category) statistically gives ~ 44 species in peripheral Makay and ~ 36 species in inner Makay, without overlap between the 95% confidence intervals (Fig. 6B). The analysis also suggests more species in non-forested areas than in forested or semi-forested ones (for a random sampling of 1000 individuals: forested, 28 species; semi-forested, 36 species; non-forested, 51 species; confidence intervals not overlapping between “non-forested” and the other two categories) (Fig. 6B). However, extrapolation for higher numbers of individuals (Fig. 6B) as well as the asymptotic analysis yielded overlapping 95% confidence intervals for H_0_ estimates between all pairs of compared categories, so that in fact these categories cannot be ranked with certainty for species richness. Estimates of H_1_ (which takes into account both species richness and abundance evenness) were more straightforward (Fig. 6C), indicating higher species diversity for peripheral sites than for inner Makay sites, and higher species diversity for sites located in non-forested environments than for sites located in arbored environments (for both comparisons, with no overlap of confidence intervals in the asymptotic analysis), but no difference between forested and semi-forested sites. Estimates using H_2_ (which put more weight on evenness than H_1_) led to the same conclusions for vegetation contexts, but no significant difference between the peripheral and inner massif areas (overlapping confidence intervals; Fig. 6D).

## ﻿Discussion

This faunistic study represents the first survey of predaceous water beetles (aquatic Adephaga) in freshwater habitats of the Makay massif and its immediate surroundings. All of the 74 sampled species except *Laccophilusmakay* are newly recorded for the study area. In line with previous studies on terrestrial taxa (see Introduction), the results highlight the considerable interest and originality of the Makay as a biodiversity sanctuary. At the same time, as we will see, the results reveal that for aquatic Adephaga, levels of species diversity and endemicity in inner Makay are comparatively and rather curiously low. Both areas of the Makay massif explored in this study (northern and central-southern sites) appear to be highly homogeneous in terms of species contingent. Notably, for inner massif sites the dominant species were the same in both areas. A few remarkable species (*Cybisteroperosus*, *Hydaticuspetitii*, and the two newly described *Copelatus*) were found in the northern area only, but we cannot exclude their presence in the central-southern area as well.

In the current state of knowledge, five species of aquatic Adephaga (all belonging to family Dytiscidae) are endemic to the Makay: *Clypeodytes* sp. Ma3 (undescribed), *Copelatusmalavergnorum* sp. nov., *Copelatuszanabato* sp. nov., *Laccophilusmakay*, and *Pachynectes* sp. Ma4 (undescribed). Although *Clypeodyes* sp. Ma3 was collected at a site located in the peripheral plain and probably exists elsewhere in western Madagascar, the four other species are more likely to be true Makay endemics as they were exclusively sampled among the canyons of inner Makay. With a relative frequency of occurrence of 65.6% and high density of individuals at many sites, *Laccophilusmakay* is by far the most abundant species of aquatic Adephaga in inner Makay, where this species can be found in virtually any kind of calm water habitat. *Pachynectes* sp. Ma4 is rarer but widespread in the massif and occasionally abundant. In contrast, the two newly described species of *Copelatus* are known from few specimens and localities, so far only in the northern part of the massif, and they seem to have more specialised ecologies (presumably semi-subterranean for *C.malavergnorum*). Other species that can be considered local endemics are those known only from the massifs of Makay and Isalo: *Africophilusbartolozzii* (described from the Isalo massif; this species appears to be abundant in some hygropetric habitats of the Makay canyons) and *Copelatusacamas* (also described and to date recorded only from the Isalo massif; one of the dominant species of Dytiscidae in the Makay canyons).

Notwithstanding this singularity and an undeniable patrimonial value, the aquatic Adephaga fauna of inner Makay is in fact rather poor. Our impression in the field when conducting samplings was that we were finding relatively few species, and almost always the same, again and again. Our data analyses showed that species diversity in inner Makay for aquatic Adephaga is lower than in the peripheral deforested lowlands, furthermore with an endemism level of only 55.3% (53.3% for the peripheral lowlands), to be compared with the global percentage of endemic species for aquatic Adephaga in Madagascar, ~ 74% (value compiled from data in Bergsten in press, Bergsten and Manuel in press; Bergsten et al. in press; Gustafson et al. in press). This relatively low relative endemism level for inner Makay is at least in part due to the presence in low numbers of many of the non-endemic species that are common in the surrounding peripheral area. The degree of this effect might constitute a difference between the drier forests of western Madagascar and the more closed-canopy humid forests of the northern and eastern parts of the country. From a more qualitative point of view, the relative poorness of the inner Makay aquatic Adephaga fauna is also exemplified by the absence (or low species number) in inner Makay for some particular taxa, known throughout Madagascar to be good indicators for well-preserved wooded habitats (discussed in Bergsten et al. in press). The Gyrinidae of inner Makay are poorly diversified with only three species in our samplings, including a single species for the genus *Orectogyrus* and noticeably no species of *Aulonogyrus*. Among Dytiscidae, three genera (*Madaglymbus*, *Hovahydrus*, and *Uvarus* – the first two being Malagasy endemic genera) are usually rich in local endemics in well-preserved forested environments in Madagascar. In Makay, we found only one widespread *Madaglymbus* species (*M.fairmairei*), no *Hovahydrus*, and no locally endemic *Uvarus* species.

There is a critical lack of published studies with comparable datasets on which to confront quantitatively species diversity and endemism level of inner Makay with those of other Malagasy massifs, in order to substantiate the conclusion that species diversity and endemism level of aquatic Adephaga in the Makay massif are relatively poor. In the Central Highlands, Ranarilalatiana (2019) sampled 46, 47, and 48 species, respectively, in the relict forest massifs of Manjakatompo-Ankaratra, Ambohitantely, and Anjozorobe-Angavo. These numbers are very close to our observed species richness for inner Makay (47); however, observed species richness depends strongly on sampling strategy and sample coverage so that these quantities are in fact not directly comparable. Interestingly, for Ambohitantely, observed species richness was higher outside the protected area boundaries than inside, which is reminiscent of the results we obtained for the Makay from interpolation-extrapolation analyses, whereas for the other two Central Highland massifs, the pattern was the opposite (Ranarilalatiana 2019).

The Makay massif bears strong similarities and a geographical proximity with the massif of Isalo, making comparison of the aquatic Adephaga fauna between these two massifs particularly appealing. The massifs of Makay and Isalo, isolated from each other by the large Mangoky River plain, are at first approximation rather similar in terms of geology (sandstone substratum) and geomorphology (deep canyons). For aquatic Adephaga, they also have strong faunistic affinities. Of the six species for which we obtained RFO>20% in inner Makay, five are also present and common in Isalo (*Copelatusacamas*, *C.ruficapillus*, *Dineutusproximus*, *Hyphydrusseparandus* and *Pachynectes* sp. Ma1). In addition to the two local endemics known only from Isalo and Makay mentioned above, a few endemics with more widespread but more or less localised distributions in Madagascar are also present in both massifs: *Cybisteroperosus*, *Laccophilustransversovittatus*, and *Neptosternusoblongus*. Furthermore, we can mention two interesting cases of local endemic vicariance between Isalo and Makay. *Laccophilusmakay* is replaced in the Isalo massif by another species of the *alluaudi*-group, *L.pseustes* Guignot, 1955, which is very abundant in habitats similar to those occupied by *L.makay* in the Makay. In Isalo, there is an undescribed species of *Pachynectes* which is morphologically very close to *P.* sp. Ma4 and has the same habitat preferences.

**Figure 7.**
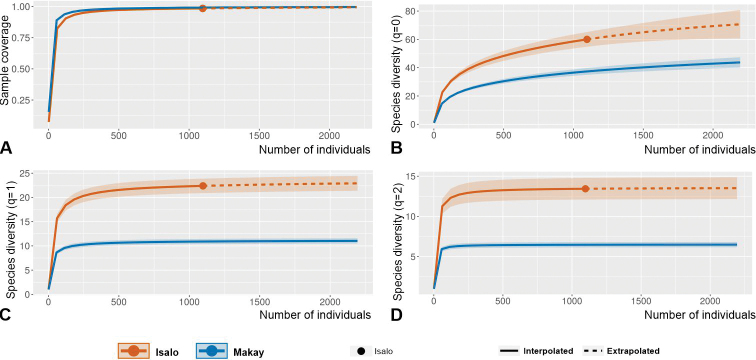
Interpolation-extrapolation graphs for inner Makay and inner Isalo. Coloured lines represent the interpolated (solid line) or extrapolated (dashed line) estimate of the metric against number of individuals; the surface of lighter colour surrounding each curve materialises the 95% confidence interval **A** sample coverage **B** Hill number of order q=0 (H_0_ or species richness) **C** Hill number of order q = 1 (H_1_) **D** Hill number of order q = 2 (H_2_).

We are able to provide comparative estimates of species diversity, based on our own unpublished sampling data in southern Isalo (obtained in May 2016, using similar collecting techniques to those deployed in the Makay; with satisfying sample coverage as shown in Fig. 7A). The following comparisons are based on samplings performed in the massif themselves (i.e., inner areas), for both Makay and Isalo (see Suppl. material 2: Table S2). The percentage of endemics among the 60 species in our sampling in inner Isalo was 60%, thus slightly higher than in inner Makay. Species richness (H_0_) appears to be higher in Isalo than in Makay according to the interpolation analysis: a random sampling of 1000 individuals statistically gives ~ 58 species in Isalo, vs. ~ 37 species in Makay, without overlap between the confidence intervals (Fig. 7B). Using the H_1_ metric, species diversity is twice higher in Isalo than in Makay (Fig. 7C); the H_1_ asymptotic analysis gives 21.61–25.05 species equivalents for Isalo, vs. 10.70–11.66 for Makay. Using the H_2_ metric, species diversity is also significantly higher in Isalo than in Makay (Fig. 7D).

Why is species diversity of aquatic Adephaga so much lower in Makay than in Isalo? Makay is much dryer than Isalo (average annual rainfall for 2009–2020 according to https://www.historique-meteo.net/: in Isalo 1485 mm, in Morombe close to the Makay 877 mm; an older reference gives 700 mm for Makay, Cornet and Guillaumet 1976), and this might be part of the explanation, but our field observations suggest additional hypotheses. The mineral substratum of inner Makay streams and rivers is almost invariably fine sand (with few or no stones, pebbles and gravel), which is not the case in Isalo. This might be due to some erosion properties of the Makay sandstone. As a consequence, in association with the strongly constraining geomorphology, the habitats available to aquatic Adephaga beetles in Makay might be characterised by a relatively high level of homogeneity and thereby low diversity of ecological niches. Another possible cause of low species diversity (but with high abundance of a few well adapted specialists) may relate to geochemistry. Slow streams and their satellite pools in Makay are very often conspicuously filled in by orange masses of iron bacteria, which may reflect peculiar geochemical characteristics of the mineral substratum. Furthermore, during field work in central-southern Makay, we were often struck by the strange smell (evoking sulphur) at places emanating from the rivers water. Studies focused on the physical characteristics of freshwater habitats and water chemistry in the Makay massif may help to assess these hypotheses. To improve knowledge on freshwater ecology of the inner Makay, it will of course also be necessary to obtain data on the diversity of other aquatic animal taxa, particularly among freshwater invertebrates. This would notably help to determine whether or not our conclusions from the study of aquatic Adephaga reflect a general trend for aquatic taxa in this area.

Finally, we would like to point out a few remarkable findings from our samplings in the peripheral plain surrounding the Makay massif. At ~ 15 km south-west of Makaikely, a small and shallow stream (MAK-2) with sandy bottom and very slowly flowing water, strongly impacted by cattle trampling and highly eutrophicated, provided an impressive sampling with 32 species of aquatic Adephaga (listed in the legend of Fig. 2B), collected in just a few square meters in ca. one hour. This included several remarkable species, such as *Laccophilusseyrigi* (first observation to our knowledge since its original description in 1937), *L.rivulosus* (a large, beautiful and rather uncommon *Laccophilus* species), *Hydrovatusdentatus* (second record for Madagascar), *H.testudinarius*, *Philaccoluselongatus*, and an undescribed species of *Clypeodytes*. Another noticeable finding, at two other peripheral sites, was *Peltodytesquadratus*, which despite being the less rare of the Malagasy Haliplidae (Van Vondel and Bergsten 2012), is nevertheless an uncommon and rather localised species. Altogether, the 12 sites located in the surroundings of the massif yielded a highly diversified and interesting set of species, showing that this largely deforested area, impacted notably by wood gathering, fires, and cattle trampling, still comprises some rich and singular elements of freshwater biodiversity.

## Supplementary Material

XML Treatment for Dineutus proximus

XML Treatment for Dineutus sinuosipennis sinuosipennis

XML Treatment for Orectogyrus vicinus

XML Treatment for Peltodytes quadratus

XML Treatment for Canthydrus concolor

XML Treatment for Canthydrus flavosignatus

XML Treatment for Canthydrus guttula

XML Treatment for Canthydrus

XML Treatment for Neohydrocoptus seriatus

XML Treatment for Neohydrocoptus

XML Treatment for Sternocanthus fabiennae

XML Treatment for Synchortus asperatus

XML Treatment for Copelatus acamas

XML Treatment for Copelatus andobonicus

XML Treatment for Copelatus polystrigus

XML Treatment for Copelatus ruficapillus

XML Treatment for Copelatus vigintistriatus

XML Treatment for Copelatus malavergnorum

XML Treatment for Copelatus zanabato

XML Treatment for Madaglymbus fairmairei

XML Treatment for Cybister cinctus

XML Treatment for Cybister operosus

XML Treatment for Rhantaticus congestus

XML Treatment for Eretes griseus

XML Treatment for Hydaticus dorsiger

XML Treatment for Hydaticus exclamationis

XML Treatment for Hydaticus petitii

XML Treatment for Hydaticus servillianus

XML Treatment for Hydaticus sobrinus

XML Treatment for Bidessus longistriga

XML Treatment for Bidessus perexiguus

XML Treatment for Clypeodytes concivis

XML Treatment for Clypeodytes insularis

XML Treatment for Clypeodytes

XML Treatment for Hydroglyphus capitatus

XML Treatment for Hydroglyphus geminodes

XML Treatment for Hydroglyphus plagiatus

XML Treatment for Liodessus luteopictus

XML Treatment for Pachynectes costulifer

XML Treatment for Pachynectes

XML Treatment for Pachynectes

XML Treatment for Pseuduvarus

XML Treatment for Uvarus betsimisarakus

XML Treatment for Uvarus rivulorum

XML Treatment for Yola costipennis

XML Treatment for Hydrovatus acuminatus

XML Treatment for Hydrovatus capnius

XML Treatment for Hydrovatus crassicornis

XML Treatment for Hydrovatus cruentatus

XML Treatment for Hydrovatus dentatus

XML Treatment for Hydrovatus otiosus

XML Treatment for Hydrovatus parvulus

XML Treatment for Hydrovatus pictulus

XML Treatment for Hydrovatus testudinarius

XML Treatment for Hydrovatus

XML Treatment for Hyphydrus separandus

XML Treatment for Hyphydrus stipes

XML Treatment for Methles

XML Treatment for Methles

XML Treatment for Africophilus bartolozzii

XML Treatment for Africophilus nesiotes

XML Treatment for Laccophilus addendus

XML Treatment for Laccophilus flaveolus

XML Treatment for Laccophilus insularum

XML Treatment for Laccophilus luctuosus

XML Treatment for Laccophilus makay

XML Treatment for Laccophilus pallescens

XML Treatment for Laccophilus posticus

XML Treatment for Laccophilus rivulosus

XML Treatment for Laccophilus seyrigi

XML Treatment for Laccophilus transversovittatus

XML Treatment for Laccophilus

XML Treatment for Neptosternus oblongus

XML Treatment for Philaccolus elongatus

## Supplementary materials

Supplementary material 1 Table S1 Data type Cumulated abundances of species per categories of sampling sites in Makay Explanation note This table gives for each category of sites and for each species the total number of specimens sampled. The second column indicates the status of the species with respect to endemism. These data were used to build the graphs of Figs 5, 6. The table is in Excel format (.xls). https://binary.pensoft.net/file/764734This dataset is made available under the Open Database License (http://opendatacommons.org/licenses/odbl/1.0/). The Open Database License (ODbL) is a license agreement intended to allow users to freely share, modify, and use this Dataset while maintaining this same freedom for others, provided that the original source and author(s) are credited.Andriamirado T. Ramahandrison, Bakolimalala Rakouth, Michaël Manuel

Supplementary material 2 Table S2 Data type Cumulated abundances of species sampled in inner Makay vs. in inner Isalo. Explanation note This table gives for each species the total number of specimens sampled in inner Makay and in inner Isalo. These data were used to build the graphs of Fig. 7. The table is in Excel format (.xls). https://binary.pensoft.net/file/764735This dataset is made available under the Open Database License (http://opendatacommons.org/licenses/odbl/1.0/). The Open Database License (ODbL) is a license agreement intended to allow users to freely share, modify, and use this Dataset while maintaining this same freedom for others, provided that the original source and author(s) are credited.Andriamirado T. Ramahandrison, Bakolimalala Rakouth, Michaël Manuel
